# Understanding Lignin Oxidation by a Two-Domain Multicopper Oxidase from *Cellvibrio japonicus*

**DOI:** 10.1021/acs.biochem.6c00183

**Published:** 2026-04-27

**Authors:** Morten Rese, Gijs van Erven, Mirjam A. Kabel, Anton A. Stepnov, Vincent G. H. Eijsink, Tina R. Tuveng

**Affiliations:** † Faculty of Chemistry, Biotechnology, and Food Science, 393285 Norwegian University of Life Sciences (NMBU), P.O. Box 5003, Ås 1433, Norway; ‡ 4508 Wageningen Food and Biobased Research, Bornse Weilanden 9, Wageningen 6708 WG, The Netherlands; § Wageningen University & Research, Laboratory of Food Chemistry, Bornse Weilanden 9, Wageningen 6708 WG, The Netherlands

## Abstract

Bacterial two-domain multicopper oxidases (2dMCOs) represent a structurally distinct class of trimeric multicopper oxidases. Differing considerably from well-characterized monomeric three-domain laccases, type B 2dMCOs have a T1 active site positioned in a tunnel in the trimer center. The biochemical properties and roles in lignin conversion of 2dMCOs remain poorly understood. Here, we present a comprehensive biochemical characterization of a type B 2dMCO from *Cellvibrio japonicus* (*Cj*MCO) and discuss links between its structural organization and activity. The T1 copper of *Cj*MCO had a redox potential of 537 mV, but the turnover number (0.4 s^–1^) was ∼1000-fold lower than high-turnover fungal laccases. Stopped-flow UV–vis spectroscopy indicated that this low turnover likely reflects slow reoxidation of the enzyme by O_2_, which has not been previously reported for laccases. Despite the steric constraints imposed by its trimeric structure, *Cj*MCO oxidized the lignin model compound guaiacylglycerol-β-guaiacyl ether, resulting in both oxidative coupling and bond cleavage, and *Cj*MCO was able to act on oligomeric birch organosolv lignin, promoting net oxidative polymerization. Interestingly, 2,6-dimethoxyphenol oxidation kinetics and the product profile for guaiacylglycerol-β-guaiacyl ether oxidation by *Cj*MCO were influenced by pH, buffer composition, and ionic strength, suggesting a potential strategy for tailoring product profiles. Together, these findings demonstrated that *Cj*MCO functions as a laccase and oxidizes phenolic lignin moieties, but its slow rates and trimeric architecture indicate that it is unlikely to efficiently degrade lignin polymers *in vivo*. This study expands the current understanding of bacterial laccase diversity and provides a foundation for exploring other physiological roles of type B 2dMCOs beyond lignin degradation.

## Graphical abstract

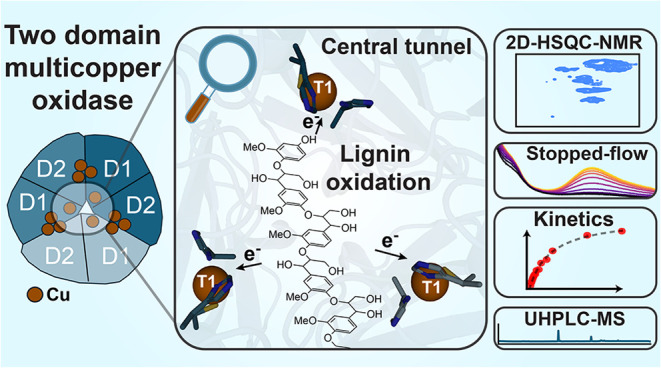

## Introduction

Multicopper oxidases (MCOs) are a diverse family of enzymes that couple oxidation of various organic and inorganic substrates to reduction of molecular oxygen to water.^1^ Laccases (EC 1.10.3.2) are a subgroup of MCOs with low substrate specificity, acting on a range of phenolic compounds, in contrast to e.g., ferroxidases and ascorbate oxidases, which are MCO subgroups with high specificity. Laccase activity is important for several biological processes,^2,3^ including degradation of lignin.^4^ Lignin is a heterogeneous, aromatic polymer that, together with cellulose and hemicellulose, forms the complex lignocellulosic matrix of secondary plant cell walls. The recalcitrant nature of lignin contributes to the resistance of lignocellulose to microbial degradation. As a result, specialized microorganisms, including bacteria and fungi, have evolved both enzymatic and nonenzymatic systems to degrade and/or modify lignin.^5^ The main enzymes known to contribute to the degradation of polymeric lignin are lignin peroxidases, manganese peroxidases and laccases.^6^ While fungal laccases, especially those expressed by white-rot fungi, have been extensively studied for their role in lignin degradation,^4^ the role of bacterial laccases in lignin degradation remain less explored.^7^

Like all MCOs, laccases catalyze one-electron substrate oxidation at a mononuclear type-1 (T1) copper site,^8^ followed by intramolecular electron transfer to a trinuclear copper cluster (TNC) consisting of two type-3 and one type-2 copper sites. After four sequential substrate oxidations, the fully reduced enzyme catalyzes the concerted four-electron reduction of molecular oxygen to water. Depending on the enzyme laccases have reduction potentials between 340 and 780 mV,^8^ which is insufficient to oxidize nonphenolic lignin units (∼80% of the subunits of the lignin polymer).^9^ In contrast, fungal lignin peroxidases have redox potentials exceeding 1000 mV, enabling oxidation of nonphenolic lignin units.^10^ Still, laccases can act directly on lignin, via oxidation of the phenolic units, generating phenoxy radicals.^11^ These radicals dissipate through nonenzymatic reactions, predominantly leading to radical coupling, but which also could lead to bond cleavage.^12^ Radical coupling produce new linkages not originally present in the native lignin, including condensed C–C bonds,^13,14^ leading to polymerization and cross-linking of lignin fragments, thereby increasing its molecular weight.^15–18^ Thus, laccase action alone is often ineffective at degrading polymeric lignin *in vitro*.^11^ A commonly used *in vitro* approach for degrading lignin by laccases involves a laccase-mediator system.^19^ In such a system, a small redox mediator is first oxidized by laccase, and the resulting species, with an increased redox potential, then oxidizes the lignin, primarily through hydrogen-atom transfer (HAT). Synthetic mediators such as 1-hydroxybenzotriazole (HBT) and 2,2′-azino-bis­(3-ethylbenzothiazoline-6-sulfonic acid) (ABTS) have been shown to facilitate lignin degradation by laccases.^20,21^ While some studies suggest that natural lignin-derived aromatic compounds can act as mediators in laccase mediator systems and thus facilitate lignin degradation,^22–26^ the supporting evidence remains limited, and it is not yet established whether such mediators play a significant role in nature. Consequently, the role of laccases in biological lignin degradation remains unclear, highlighting the need for further characterization of these enzymes.

Structurally, MCOs are categorized by the number and organization of their cupredoxin-like domains and the ability of the domains to bind copper. MCOs contain either two (2dMCO), three (3dMCO), or six (6dMCO) cupredoxin-like domains.^27^ 2dMCOs are found in bacteria and archaea, 3dMCOs are present in both bacteria and fungi (and are the predominant type in fungi), while 6dMCOs are rare and occur in select bacterial and fungal lineages. Both 3dMCOs and 6dMCOs occur as monomeric proteins, while 2dMCOs form trimeric structures, with residues from two protomers contributing to coordination of the coppers at the TNC-site. Classification of 2dMCOs is based on the number and location of the T1 copper site in one of the protomers: either in the C-terminal cupredoxin domain (type B), the N-terminal cupredoxin domain (type C), or in both domains (type A).^27^

Most laccases characterized in the context of lignin degradation are fungal 3dMCOs, particularly those from white-rot fungi.^28,29^ In contrast, 2dMCOs are far less studied. It remains unclear what fraction of 2dMCOs are *bona fide* laccases and how effectively they oxidize lignin. Although a few 2dMCOs have been characterized, such as type B “small laccase” (SLAC) enzymes from *Streptomyces sviceus* and *Streptomyces coelicolor*, where the latter was shown to be capable of oxidizing lignin,^30,31^ our understanding of 2dMCOs remains limited. Sequence-based analyses further subdivide type B 2dMCOs into at least two distinct sequence superfamilies: superfamily K, comprising SLAC-like enzymes, and superfamily N, encompassing other bacterial type B 2dMCOs.^27^ The physiological roles of these different 2dMCOs, and particularly their potential contribution to lignin degradation, are poorly understood, especially for superfamily N 2dMCOs.

In this study, we present a detailed biochemical characterization of a type B 2dMCO from superfamily N, encoded as the sole 2dMCO in the genome of the lignocellulose-degrading soil bacterium *Cellvibrio japonicus*. Since this bacterium encounters lignin-rich biomass in its natural habitat, this enzyme provides a unique opportunity to assess whether bacterial 2dMCOs can function as laccases and participate in lignin modification. Because *C. japonicus* is a well-studied model for bacterial lignocellulose utilization,^32^ elucidating the role of its single 2dMCO provide insight into whether 2dMCOs play a functional role in bacterial lignin modification.

## Materials and Methods

### Materials

Unless otherwise stated, all chemicals were purchased from Sigma-Aldrich (St. Louis, MO, USA). All buffers were prepared with ultrapure water (resistivity ≥ 18.2 MΩ cm) using a Milli-Q system (Millipore, Billerica, MA, USA). HPLC-grade solvents for chromatographic and mass spectrometric analyses were purchased from VWR (Radnor, PA, USA).

### Protein Structure Modeling

The three-dimensional model structure of the homotrimeric complex of *Cj*MCO was obtained by utilizing AlphaFold 3.^33^ Three copies of the mature amino acid sequence (*Cj*MCO with the signal peptide, residue 1–24, removed) and in total 12 copper atoms (four per *Cj*MCO subunit) were given as input to AlphaFold to generate the structure. PyMOL 4.6.0 ^34^ was used to visualize protein structures.

### Phylogenetic Analysis

For the generation of the phylogenetic tree of bacterial multicopper oxidases, 1240 sequences from Ausec et al.^35^ were used. Multiple sequence alignment was performed using MAFFT^36^ with default settings and the phylogenetic tree was built using FastTree.^37^

### Expression and Purification of *Cj*MCO

A codon-optimized gene (GenScript, Piscataway, NJ, USA) encoding the mature multicopper oxidase (referred to as *Cj*MCO) from *Cellvibrio japonicus* (residue 25–468, UniProt ID: B3PHM6) was PCR-amplified and cloned into a pNIC–CH plasmid using ligation-independent cloning without adding the C-terminal His-tag encoded by the plasmid. A truncated variant of the same gene (referred to as *Cj*MCOΔ25–66 and containing residue 67–468 and removing a fragment encoding the His-rich N-terminal region of the protein) was also constructed. Correct DNA sequence was confirmed by Sanger sequencing before the pNIC–CH plasmids were transformed into BL21 (DE3) competent *Escherichia coli* cells (Thermo Scientific, Waltham, MA, USA) using the heat shock method.

Production of the *Cj*MCO and *Cj*MCOΔ25–66 proteins was performed by inoculating 0.5 L Terrific Broth containing 50 mg/L kanamycin and 0.015% Antifoam 204 (Sigma-Aldrich) with a 5 mL overnight culture. The culture was incubated at 37 °C and gene expression was induced by adding isopropyl β-D-1-thiogalactopyranoside to a final concentration of 0.2 mM at OD_600_ ∼ 0.6. After induction, the culture was incubated at 30 °C for 16–20 h with high oxygenation using a LEX-48 Bioreactor (Harbinger biotech, Markham, Canada) before harvesting cells by centrifugation.

Harvested cells were resuspended in 1/20 culture volume of a lysis buffer (500 mM NaCl, 20 mM imidazole, 50 mM TRIS-HCl, pH 7.5, 0.1 g/L lysozyme, and 0.1 U/mL DNase I (Thermo Scientific). The lysis buffer for *Cj*MCOΔ25–66 did not contain NaCl and imidazole. Phenylmethylsulfonyl fluoride (PMFS) was added to 0.01 mM and the cells were lysed by sonication at 29% amplitude with a pulse of 5 s on, 5 s off. Subsequently, cell debris was removed by centrifugation at 20 000 x *g* for 20 min at 4 °C. The supernatant was filtered (0.22 μM) prior to protein purification.

*Cj*MCO was purified by nickel affinity chromatography using a 5 mL HisTrap column (Cytiva, Marlborough, MA, USA) with 0.5 M NaCl, 20 mM imidazole, 50 mM TRIS-HCl, pH 7.5, as mobile phase. Bound proteins were eluted using a 20–400 mM imidazole linear gradient over 100 mL at 3 mL/min. *Cj*MCO, with its native histidine-rich N-terminal region, can bind to the Ni-resin in the HisTrap column without a conventional HisTag.

*Cj*MCOΔ25–66 was purified by anion exchange chromatography using a 5 mL HiTrap DEAE FF column (Cytiva) with 50 mM TRIS-HCl, pH 7.5, as mobile phase. The protein mixture was loaded at 1 mL/min and bound proteins were eluted using a linear gradient from 0 to 300 mM NaCl over 150 mL at 3 mL/min.

The presence and purity of proteins in the eluted fractions were evaluated by SDS-PAGE electrophoresis. Fractions containing purified enzyme were pooled, concentrated, and buffer exchanged to 50 mM TRIS-HCl, 0.2 mM NaCl, pH 7.5, using 30 kDa cutoff Amicon Ultra-15 centrifugal filters (Merck, Darmstadt, Germany). *Cj*MCO and *Cj*MCOΔ25–66 were then subjected to a second purification step by size-exclusion chromatography using a ProteoSEC Dynamic 3–70 kDa HR (Protein Ark, Sheffield, UK) column with 50 mM TRIS-HCl, pH 8.0, 200 mM NaCl as the running buffer. Fractions containing *Cj*MCO or *Cj*MCOΔ25–66 were pooled, concentrated, and buffer exchanged to 50 mM MOPS, pH 7.0, as described above. Protein concentrations were determined using the theoretical extinction coefficient at 280 nm (64 400 M^–1^ cm^–1^ for both variants).

### Native Polyacrylamide Gel Electrophoresis (PAGE)

Native PAGE was conducted using 4 μg of protein sample loaded onto an Any kD Mini-PROTEAN TGX Precast Gel (Bio-Rad, Hercules, CA, USA). Electrophoresis was performed at 100 V for 100 min in NativePAGE Running Buffer (Invitrogen, Carlsbad, CA, USA). NativeMark Unstained Protein Standard (Invitrogen) was used as a standard.

### Copper Saturation

Copper saturation was performed as previously described.^38^ In brief, a 1 mM CuSO_4_ solution in 50 mM MOPS, pH 7.0, with 1 mM reduced glutathione was titrated into a 50–100 μM *Cj*MCO or *Cj*MCOΔ25–66 solution, reaching a final ratio of 20 mol equivalents of copper to *Cj*MCO and incubated for 30 min at 4 °C. Unbound copper was removed by excessive dilution and concentration with 50 mM sodium phosphate buffer (pH 7.0) using 30 kDa cutoff Amicon Ultra-15 centrifugal filters until the theoretical final concentration of unbound copper was <1 nM. All further experiments were performed within 24 h following copper saturation. Hereafter, copper-saturated *Cj*MCO is referred to as *Cj*MCO, whereas the protein used directly after purification without copper saturation is referred to as *Cj*MCO^as is^.

### Copper Quantification

The 2,2-biquinoline assay was performed to quantify copper in *Cj*MCO and *Cj*MCOΔ25–66 as previously described.^39^ A 0.5 g/L 2,2-biquinoline solution was prepared in glacial acetic acid. Assays were carried out in quartz cuvette in a final volume of 1.0 mL containing 600 μL 2,2-biquinoline solution, 20 μL saturated ascorbic acid, and either 380 μL 50 mM MOPS buffer (pH 7.0) for blanks or 100 μL protein and 280 μL MOPS buffer for protein measurements. Absorbance was measured at 546 nm with a Cary 60 UV–vis spectrophotometer (Agilent Technologies, CA, USA). Measurements were performed in triplicate, and copper concentration was calculated from the blank-corrected absorbance using ε_546 nm_ = 6300 M^–1^ cm^–1^.

### Preparation of Laccase from *Trametes versicolor* (*Tv*Lac)

A crude extract containing *Tv*Lac (Sigma-Aldrich) was dissolved in 50 mM sodium phosphate, pH 6.0. The sample was 0.22 μm filtered and then applied to a HiTrap 5 mL DEAE FF column followed by elution using a gradient of 0–1000 mM NaCl in 50 mM sodium phosphate, pH 6.0. The presence and purity of *Tv*Lac in eluted fractions were evaluated by SDS-PAGE and fractions containing high-purity *Tv*Lac were pooled and concentrated using 30 kDa cutoff Amicon Ultra-15 centrifugal filters.

### Determination of the Reduction Potential

The reduction potential of the T1-Cu of *Cj*MCO was determined through poised potential titration at 25 °C, using the K_3_[Fe­(CN)_6_]/K_4_[Fe­(CN)_6_] redox couple. A value of +425 mV vs the standard hydrogen electrode was used for 100 mM sodium phosphate buffer at pH 7.0.^40^ Measurements were performed in an anaerobic chamber using a NanoPhotometer C40 (Implen, Munich, Germany). Buffer and enzyme solutions were degassed by flushing with N_2_ and transferred to the anaerobic chamber. K_3_[Fe­(CN)_6_] and K_4_[Fe­(CN)_6_] were transferred dry into the anaerobic chamber. The titration mixture consisted of 120 μL (total volume) of 50 μM *Cj*MCO in 100 mM sodium phosphate buffer, pH 7.0, supplemented with 20.8 mM K_3_[Fe­(CN)_6_], ensuring that the enzyme was initially fully oxidized. Redox titrations were initiated by stepwise addition of a concentrated K_4_[Fe­(CN)_6_] solution (5 mM). After each addition, the solution was equilibrated, and the absorbance spectrum was recorded. The redox state of the T1-Cu site was monitored by the absorbance at 603 nm, which is characteristic of the oxidized Cu­(II) form. Titration continued until complete reduction of the T1-Cu site. During the titration, the concentrations of K_3_[Fe­(CN)_6_] and K_4_[Fe­(CN)_6_] varied from 20.3 to 18.5 mM and from 0.13 to 0.67 mM, respectively. The ratio of oxidized to reduced T1-Cu, log­([Cu­(II)]/[Cu­(I)]), was calculated from the normalized absorbance at 603 nm. The solution redox potential at each titration point was calculated using the Nernst equation based on the known concentrations of K_3_[Fe­(CN)_6_] and K_4_[Fe­(CN)_6_]. A linear least-squares fit of the solution potential versus log­([Cu­(II)]/[Cu­(I)]) was performed and the reduction potential of the T1-Cu was obtained at log­([Cu­(II)]/[Cu­(I)]) = 0. Average values and standard deviations were calculated from three independent experiments.

### Stopped-Flow UV–Vis Absorbance Spectroscopy

Experiments were carried out using an SFM-4000 stopped-flow instrument (BioLogic, Grenoble, France) equipped with a TIDAS S 500 MCS UV/NIR 1910 diode array (J&M Analytik AG, Essingen, Germany). All measurements were conducted under anaerobic conditions. Buffers and *Cj*MCO solutions were purged with N_2_ gas prior to transfer into a Whitley A95TG anaerobic glovebox (Don Whitley Scientific, West Yorkshire, UK). A concentrated *Cj*MCO stock solution was degassed by N_2_ purging and equilibrated inside the anaerobic chamber before dilution with anaerobic buffer to working concentration. Hydroquinone was transferred into the glovebox in solid form, and anaerobic stock solutions were prepared inside the chamber prior to experiments. All experiments were performed at 25 °C in 100 mM sodium phosphate, pH 7.0, unless stated otherwise. Before starting the experiments, the sample lines in the stopped-flow instrument were flushed with a 10 mM sodium dithionite solution and extensively rinsed with deoxygenated buffer to eliminate any residual O_2_. To monitor *Cj*MCO reduction, ∼200 μM enzyme (premixing concentration) was mixed 1:1 (v/v) in the stopped-flow with 20 mM hydroquinone at 25 °C. For oxidation experiments, fully reduced *Cj*MCO was prepared by treating the resting (i.e., oxidized) enzyme with excess ascorbic acid, followed by buffer exchange using a PD-10 column inside the anaerobic chamber to remove the reductant. A saturated O_2_ solution (1.2 mM) was prepared by flushing a 100 mM sodium phosphate, pH 7.0, solution with gaseous O_2_. The concentration of O_2_ was determined with a OXY-4 micro Multichannel Micro Fiber Optic Oxygen Meter (PreSens, Regensburg, Germany). A 1 mM O_2_ solution was prepared by mixing the saturated O_2_ solution with degassed buffer. When performing the reaction in the stopped-flow, the reduced enzyme was mixed 1:5 with a 1 mM O_2_ solution giving final concentration of reduced *Cj*MCO and O_2_ 100 μM and 500 μM respectively.

### ABTS, Fe^2+^, and 2,6-Dimethoxyphenol (2,6-DMP) Oxidation Kinetics

For all kinetic experiments, reactions were initiated by adding *Cj*MCO to a final concentration of 1 μM into buffer containing substrates at concentrations ranging from 0–5 mM that had been pre-equilibrated at 25 °C. Reaction progress was monitored and initial reaction rates were determined from the initial linear part of the progress curve (up to 30 seconds). Oxidation of ABTS to the ABTS radical cation (ABTS^+^•) was carried out in 100 mM sodium acetate buffer, pH 5.5, in a total reaction volume of 1 mL using a quartz cuvette. Formation of the colored ABTS^+^• radical was monitored with a Cary 60 UV–Vis spectrophotometer (Agilent Technologies, CA, USA) at 436 nm (ε_436 nm_ = 29 300 M^–1^ cm^–1^). Oxidation of Fe^2+^ to Fe^3+^ was performed using (NH_4_)_2_Fe­(SO_4_)_2_ as a substrate. Formation of Fe^3+^ was observed at 315 nm (ε_315 nm_ = 2200 M^–1^ cm^–1^) in a 1 mL quartz cuvette in 100 mM MES, pH 6.5, using a Cary 60 UV–Vis spectrophotometer. Oxidation of 2,6-DMP was assessed using 96-well microplates (Thermo Scientific) with a final reaction volume of 200 μL per well. The oxidation of 2,6-DMP to coerulignone was monitored spectrophotometrically using a Varioskan LUX Multimode Microplate Reader (Thermo Scientific) by measuring the increase in absorbance at 469 nm, and using an extinction coefficient of 49,600 M^–1^ cm^–1^.^41^ Kinetic parameters (*K*_m_ and *V*_max_) were determined by fitting the initial reaction rate versus substrate concentration to the Michaelis–Menten equation. All measurements were performed in triplicate, and data are reported as mean ± standard deviation.

For comparative analysis of catalytic activity among *Cj*MCO, *Cj*MCO^as is^, *Cj*MCOΔ25–66, and *Cj*MCOΔ25–66^as is^, apparent turnover frequencies were determined at a single, fixed substrate concentration under identical conditions. These measurements were conducted in 100 mM potassium phosphate buffer, pH 6.0, using final concentrations of 0.5 mM ABTS or 10 mM 2,6-DMP.

### UV–Vis Spectroscopy of *Cj*MCO and *Cj*MCO^as is^

UV–vis spectroscopy was performed with a Cary 60 UV–Vis spectrophotometer. UV–vis spectra of *Cj*MCO and *Cj*MCO^as is^ were recorded from 300 to 750 nm at a protein concentration of 60 μM in 50 mM MOPS, pH 7.0, in a quartz cuvette with a path length of 1 cm.

### Preparation of Birch Organosolv Lignin

Organosolv lignin was prepared as previously described.^42^ Briefly, debarked birch wood was knife-milled to <2 mm particle size and subjected to organosolv extraction in a 600 mL stirred high-pressure reactor using 50 wt % aqueous ethanol and a biomass loading of 10 wt %. The suspension was kept at 190 °C for 90 min. Then, the slurry was separated using a hydraulic press and the liquid fraction was vacuum filtered. Lignin was precipitated by diluting the liquid fraction 1:4 (w/w) with deionized water and stirring at room temperature for 2 h. The suspension was centrifuged at 12 000 × *g* for 30 min, and the lignin pellet was dried at 45 °C for at least 48 h.

### *Cj*MCO Reactions with Birch Organosolv Lignin as Substrate

Reactions containing 2.5 g/L organosolv lignin (from a 50 g/L stock solution, initially dissolved in 50% v/v acetonitrile), 10 mM sodium phosphate buffer, pH 7.0, and 5 μM *Cj*MCO or *Cj*MCO^as is^ (i.e., not copper saturated) were performed in 50 mL polypropylene tubes (Greiner Bio-One, Kremsmünster, Austria) at 25 °C with shaking at 150 rpm for 18 h. A control reaction replacing enzyme with 30 μM CuSO_4_ was also performed under otherwise identical conditions. All reactions were done in triplicates. Triplicates were pooled and freeze-dried before analysis with SEC and HSQC NMR.

### ^1^H–^13^C HSQC NMR Spectroscopy of Organosolv Lignin Reactions

^1^H–^13^C HSQC NMR measurements were performed as reported previously^43^ by dissolving approximately 30 mg freeze-dried organosolv reaction mixture in 0.6 mL DMSO-*d*_6_. Measurements were performed on a Bruker AVANCE III 600 MHz NMR spectrometer (Bruker BioSpin, Rheinstetten, Germany) equipped with a 5 mm cryo-probe located at MAGNEFY (MAGNEtic resonance research FacilitY, Wageningen, The Netherlands). ^1^H–^13^C HSQC NMR spectra were recorded and processed as reported.^43^

Semiquantitative analysis of the HSQC volume integrals was performed as previously described,^44^ making use of the chemical shifts reported in the literature for annotation.^45^ S_2,6_, and G_2_ signals were used for quantifying the abundance of S and G units, respectively, where the S_2,6_ volume integral was logically divided by two. The occurrence of oxidized analogues (G_ox_ and S_ox_) was estimated in a similar manner. In the aliphatic oxygenated region, β-*O*-4 aryl ether substructures and their C_α_-ethoxylated and C_α_-oxidized analogues were quantified based on their C_β_-H_β_ correlations. For β-5 phenylcoumaran, β–β resinol and β–β epiresinol substructures, their respective C_α_-H_α_ correlations were used. Volume integrals for β–β resinol substructures were adjusted by halving. Cinnamyl alcohol substructures were estimated from their C_γ_-H_γ_ correlations and volume integrals were halved. In the aldehyde region, cinnamaldehyde, β-*O*-4-linked cinnamaldehyde and benzaldehyde substructures were estimated from their respective C_γ_-H_γ_ and C_α_-H_α_ correlations. Volume integration of all signals was performed at equal contour levels, with the integrals normalized to the size of the −OCH_3_ signal. The abundance of each lignin unit was then calculated as a percentage of the total lignin subunits, i.e., G + G_ox_ + S + S_ox_. The percentage abundance was expressed per 100 aromatic rings.

### SEC of Organosolv Lignin

Alkaline SEC was performed as described by Constant et al. (Method D).^46^ Briefly, lignin was dissolved in 0.5 M NaOH (eluent) in a concentration of 1 mg/mL and separated by using two TSKgel GMPWxl columns (7.8 × 300 mm, particle size 13 μm) in series equipped with a TSKgel guard column PWxl (6.0 × 40 mm, particle size 12 μm). The elution of analytes was monitored by measuring absorption at 280 nm. Sodium polystyrenesulfonate (PSS) standards and phenol were used for calibration. Protobind 1000 lignin (Wheat straw/Sarkanda grass soda lignin, GreenValue S.A, Orbe, Switzerland), was used as reference standard.

### Reactions with Guaiacylglycerol-Beta-Guaiacyl Ether (GBG)

All reactions with GBG as substrate were performed in 1.5 mL Eppendorf tubes containing 200 μM GBG and 1 μM enzyme. Reactions comparing *Cj*MCO with *Cj*MCO^as is^ were performed in 50 mM sodium phosphate, pH 7.0. A control reaction replacing enzyme with 4 μM CuSO_4_ was included. Reactions were incubated in a Thermomixer (Eppendorf) at 40 °C, 1000 rpm for 2 or 24 h.

Reactions aimed at determining depletion of GBG at different pH values over time were performed in a combined buffer containing 20 mM sodium acetate, 20 mM sodium phosphate, and 20 mM Tris-HCl adjusted to different pHs (5.0, 6.0, 7.0, 8.0, and 9.0). Reactions were incubated in a Thermomixer (Eppendorf) at 30 °C, 1000 rpm, and samples were withdrawn at various time points. To stop the reactions, sodium azide was added to a final concentration of 1 mM. Reactions were filtered through a 0.45 μm filter before UHPLC-MS or MALDI-TOF MS analysis. All reactions were done in triplicates.

### Ultrahigh-Performance Liquid Chromatography Mass Spectrometry (UHPLC-MS)

A volume of 10 μL of each sample was injected into an Agilent (Santa Clara, CA, USA) ZORBAX Eclipse Plus C18 column (2.1 × 150 mm, 1.8 μm particle size) mounted in a Dionex UltiMate 3000 UHPLC system (Thermo Scientific). Chromatographic separation was performed at a flow rate of 0.5 mL/min using water (A) and acetonitrile (B), both acidified with 0.1% (v/v) acetic acid, as eluents. The elution profile was as follows: 0.0–32.0 min, linear gradient from 5% to 45% B; 32.0–32.1 min, linear gradient from 45% to 99% B; 32.1–34.0 min, isocratic at 99% B; 34–34.1 min, linear gradient from 99% to 5% B; 34.1–37 min, isocratic at 5% B. Eluting compounds were detected by UV absorbance at 280 nm and GBG was quantified based on a standard curve.

For mass spectrometric analysis of products, the UHPLC system was coupled to a Velos Pro dual-pressure linear ion trap mass spectrometer equipped with an electrospray ionization source (Thermo Scientific). The source capillary and heater temperatures were set to 275 °C and 200 °C, respectively, with a source voltage of 3.0 kV. Data was acquired in negative ionization mode, and MS^2^ was performed to confirm compound identities. Fragmentation spectra were collected using collision-induced dissociation with data-dependent acquisition. Data analysis was performed using Xcalibur 4.5 (Thermo Scientific).

### Matrix-Assisted Laser Desorption/Ionization Time-of-Flight Mass Spectrometry (MALDI-TOF-MS)

For MALDI-TOF-MS analysis, sample preparation involved mixing 1 μL sample with 1 μL of a 2,5-dihydroxybenzoic acid matrix solution (20 g/L in 50% acetonitrile) onto a Bruker (Billerica, MA, USA) MTP 384 ground steel target plate, followed by air-drying. Mass spectra were obtained on a Bruker Ultraflex MALDI-TOF/TOF mass spectrometer in positive mode, equipped with a 337 nm nitrogen laser, using the Bruker FlexAnalysis software (version 3.4) for data acquisition.

## Results and Discussion

### *Cj*MCO Has a Trimeric Structural Organization with a Histidine-Rich N-Terminal Region

The sequence of *Cj*MCO encodes a protein containing 468 amino acids, beginning with a 24-amino-acid signal peptide targeting the twin-arginine translocation pathway (Figure 1A), suggesting that *Cj*MCO is either a periplasmic or an extracellular protein. Residues 25–66 of the mature protein is a histidine-rich region predicted to be flexible and structurally disordered. The first 37-residues of this region contain 13 histidine residues, representing an unusually high density of histidines in such a short sequence. The presence of such histidine-rich regions has been shown in other proteins to play critical roles in metal binding and trafficking. For instance, a histidine-rich N-terminal domain in Cu,Zn-superoxide dismutases from pathogenic bacteria functions as a high-affinity metal binding site that facilitates recruitment of copper and subsequent transfer to the active site.^47^ Likewise, a C-terminal histidine-rich region in the heavy metal transport protein *At*HMA4, from *Arabidopsis thaliana*, is essential for metal binding and translocation,^48^ while a C-terminal histidine-rich region in mycobacterial GroEL1 mediates Cu­(II) binding.^49^ Although a detailed functional characterization of this region was beyond the scope of the present study, its possible contribution to copper binding and catalytic activity was investigated by generating a truncated mutant of *Cj*MCO (*Cj*MCOΔ25–66) lacking the entire histidine-rich flexible region. Copper quantification showed that full-length *Cj*MCO contained six copper ions per protomer, whereas *Cj*MCOΔ25–66 contained four copper ions per protomer (Table S1). These results indicate that *Cj*MCOΔ25–66 retains the four copper ions required for catalysis, while full-length *Cj*MCO binds two additional copper atoms, most likely in the histidine-rich region. This suggests that histidine-rich N-terminal region of *Cj*MCO may function in copper binding and possibly, copper delivery to the T1 and TNC sites. Such a histidine-rich region has, to our knowledge, not been reported in other MCOs and may represent a unique adaptation in certain MCOs of the *Cellvibrio* genus. Indeed, among the 159 type B 2dMCOs listed by Ausec et al.,^35^ only the sequence from *C. japonicus* (which is the only 2dMCO sequence from the *Cellvibrio* genus in the data set) contains such a histidine-rich region. Expanding this analysis to the MCO sequences available (11 3dMCOs and 19 2dMCOs) in the ClusteredNR database from the *Cellvibrio* genus revealed that 14 harbored similar regions. These regions generally contained fewer (4–7) histidines than found in *Cj*MCO and were exclusively found in 2dMCOs (Figure S1).

**Figure 1:**
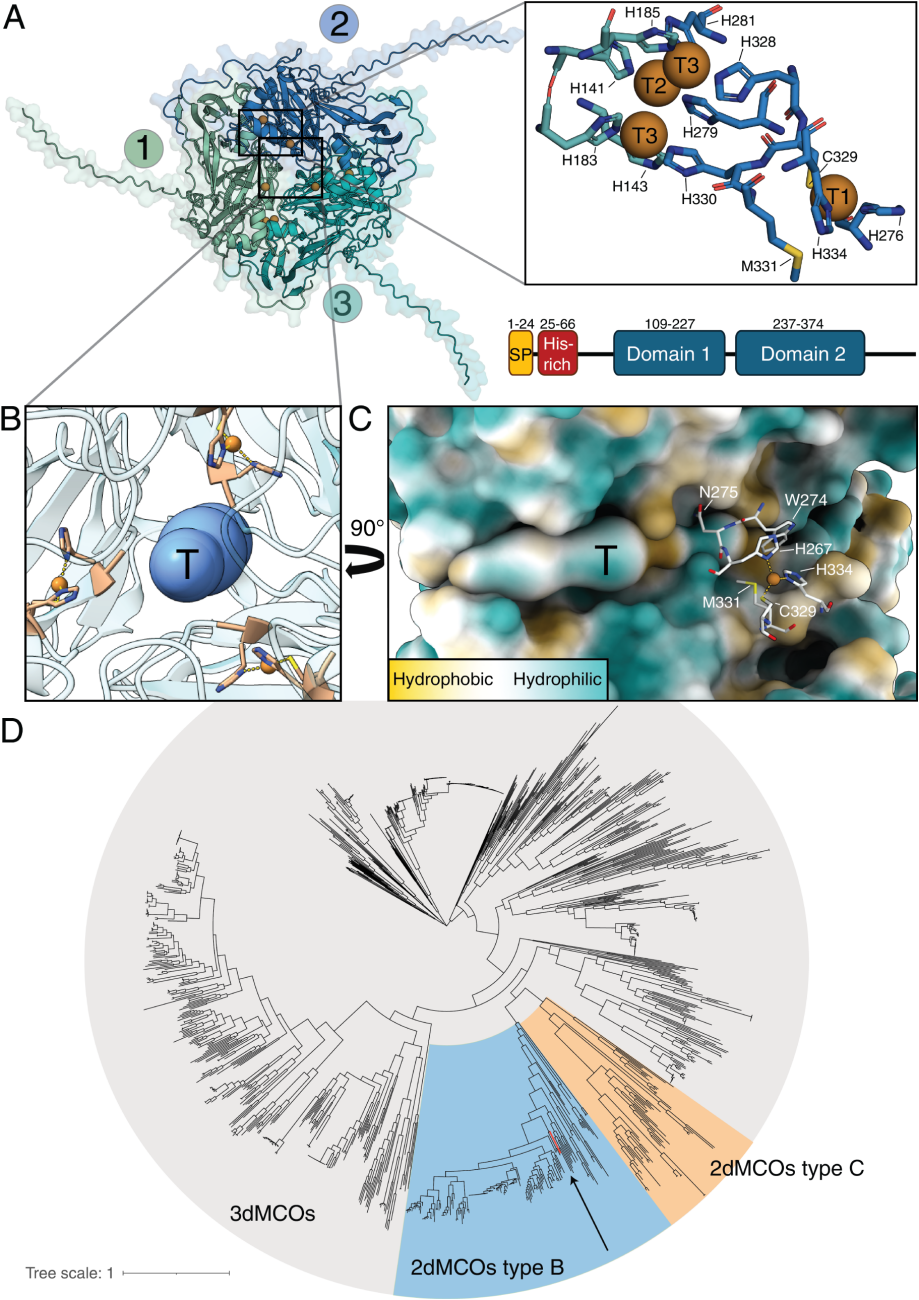
AlphaFold structure, domain organization, and phylogeny of *Cj*MCO. **(A)** The AlphaFold structure of the homotrimeric *Cj*MCO complex, containing three identical subunits with the flexible N-terminal region, is visualized as a combination of cartoon and surface representation. The three individual protein subunits are shown in distinct shades of green and blue, and numbered 1–3. The inset shows a zoomed in view of one of the three the active sites, showing the T1, T2, and T3 copper atoms where the T2 and T3 coppers (TNC copper site) are coordinated by residues from subunit 1 and 2, colored cyan and blue, respectively. Residues coordinating the copper atoms are labeled. The pTM and ipTM values for this model were both 0.86. All copper atoms and residues coordinating copper atoms had a pLDDT score >90 (Figure S3). The domain organization of a single *Cj*MCO polypeptide, featuring an N-terminal TAT signal peptide (SP, residues 1–24, not included in the AlphaFold structure), a N-terminal histidine-rich region spanning residues 25–66, and two multicopper oxidase domains (Domain 1 and Domain 2, spanning residues 109–227 and 237–374, respectively) is shown below the inset. (B) Close-up view showing the orientation of the three T1 copper sites toward a central tunnel (labeled “T”) located at the core of the trimeric enzyme. The tunnel surface, as calculated by MOLEonline,^51^ is shown in light blue. (C) The central tunnel rotated 90° relative to the view in panel B. The T1 copper site is shown together with residues W274 and N275, which are oriented toward the tunnel (labeled “T”) and are positioned closer to the tunnel than the T1 copper center. The tunnel surface and the protein are colored by hydrophobicity (yellow, most hydrophobic; blue, most hydrophilic). (D) Phylogenetic tree of 1200 bacterial MCO sequences extracted from Ausec et al.^35^ Type B and type C 2dMCOs are highlighted in blue and orange, respectively. Branches shown with a gray background represent 3dMCOs. The branch corresponding to *Cj*MCO is marked in red and indicated by an arrow. The multiple sequence alignment was performed using MAFFT,^36^ and the phylogenetic tree was built using FastTree.^37^

Type B 2dMCOs are known to occur in a homotrimeric form^27^ and the homotrimeric nature of *Cj*MCO was confirmed through native PAGE, clearly showing bands of a molecular weight consistent with formation of a trimer (Figure S2). Truncation of the flexible histidine-rich N-terminal region had no effect on trimer formation, regardless of whether the protein was “as is” after purification (i.e., no copper saturation) or reconstituted with copper. Structural modeling using AlphaFold, with four copper atoms per protein molecule was consistent with this predicted trimeric organization (Figure 1A, Figure S3). Importantly, this trimeric arrangement is unusual among well-characterized laccases^28,50^ and places the T1 sites in a distinctive structural context (Figure 1B and C). The T1 copper sites are oriented toward a central tunnel of the trimeric complex, while the TNC is coordinated by histidines in the first domain of one monomer and the second domain of an adjacent monomer. The T1 copper is coordinated by His276, His334, Cys331 and a weakly bound Met331, a configuration typical of blue copper centers.^1^ The location of the T1 copper site coordinating residues in the second domain aligns with the classification of *Cj*MCO as a Type B 2dMCO,^27^ a conclusion further supported by phylogenetic analysis that revealed its clustering with other Type B 2dMCOs (Figure 1D).

We then analyzed whether this unique structural framework leaves the enzyme with laccase-like redox properties. Redox titrations revealed a reduction potential of 537 ± 7 mV for the T1 copper in *Cj*MCO (Figure S4), which expectedly is lower than redox potential seen in three-coordinate T1 sites lacking the axial copper-coordinating methionine.^52,53^ However, the redox potential of *Cj*MCO was higher than what is usually found for other MCOs with a methionine ligand, such as *Bacillus subtilis* CotA (455 mV).^54^ Although the underlying basis for this higher potential is unclear, it clearly distinguishes *Cj*MCO from MCOs with similar T1 sites and should likely be attributed to effects from second-sphere residues.^8^ Across the whole MCO family, T1 reduction potentials falls in the range of 340–780 mV,^8^ meaning that *Cj*MCO is in the medium-potential category.^53^ MCOs with redox potentials in this range often exhibit broad substrate specificity,^55^ suggesting that *Cj*MCO is likely capable of oxidizing a diverse set of compounds.

### *Cj*MCO Reduction and Reoxidation Kinetics

To investigate how the structural and redox properties of *Cj*MCO affect catalysis, we examined the kinetics of copper reduction and reoxidation in *Cj*MCO using stopped-flow UV–vis spectroscopy. The UV–vis spectrum of the resting oxidized (RO) state of *Cj*MCO displayed spectral features characteristic of laccases (Figure 2A, Figure S5).^56^

**Figure 2:**
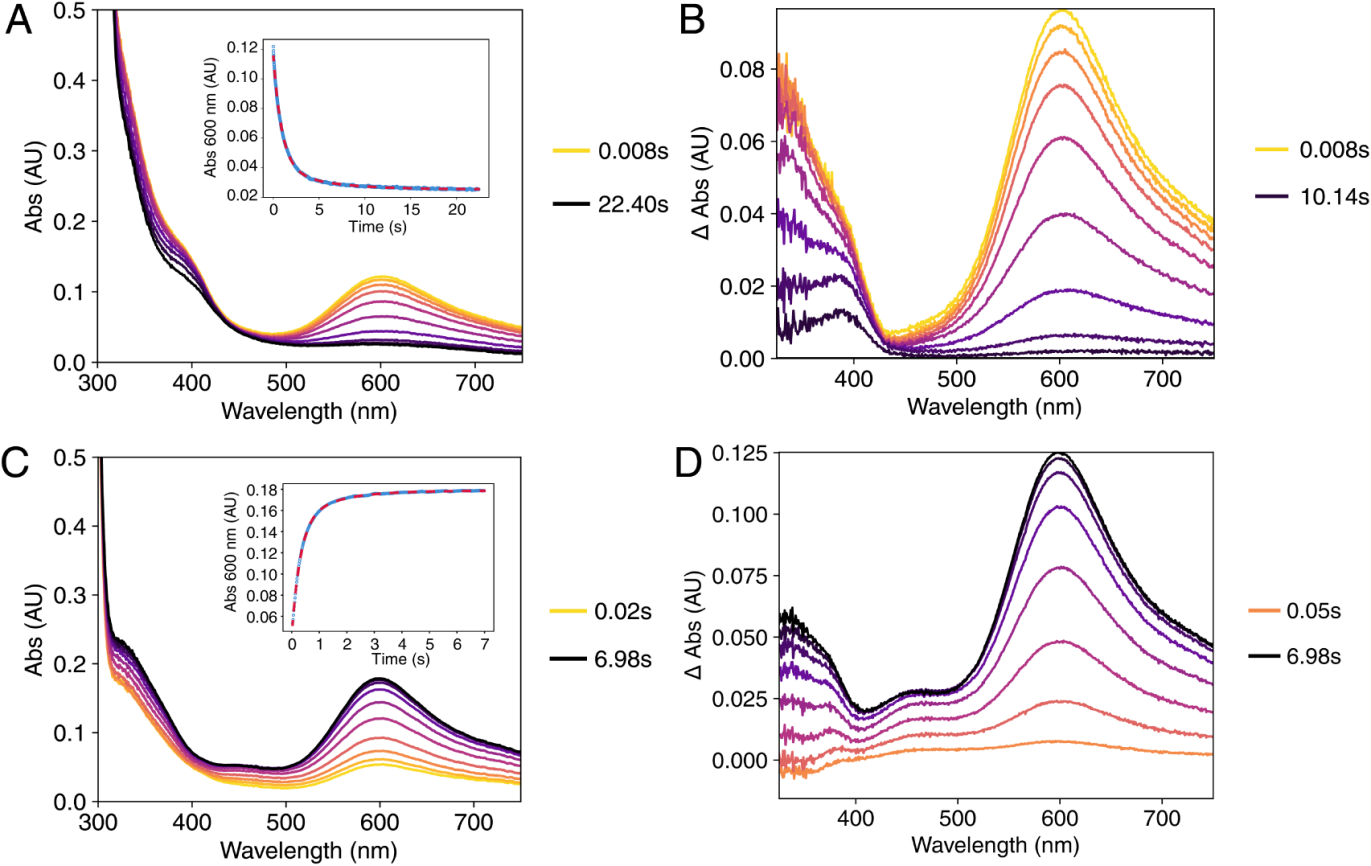
Stopped-flow UV–vis spectroscopy of *Cj*MCO reduction and oxidation. (A) Reduction of ∼100 μM *Cj*MCO by 10 mM hydroquinone in 100 mM sodium phosphate, pH 7.0, at 25 °C. The inset shows the change in absorbance at 600 nm over time (blue circles), with a fit to a double exponential function shown as a dashed red line. (B) Difference spectra illustrating the time course of reduction, generated by subtracting the spectrum of the fully reduced protein from the other spectra displayed in panel A. (C) Oxidation of ∼100 μM *Cj*MCO with 500 μM O_2_ in 100 mM sodium phosphate, pH 7.0. The inset shows absorbance at 600 nm over time (blue circles), with a double exponential fit shown as a dashed red line. (D) Difference spectra illustrating the time course of oxidation, generated by subtracting the spectrum of fully reduced protein from the spectra shown in panel C. In all panels, each spectrum represents individual time points after mixing, with the first and last time point shown in the legends. All experiments were done in triplicates at 25 °C.

The reduction and oxidation experiments showed changes in the absorption peak at 600 nm, corresponding to the redox state of the T1 copper site. However, the extinction coefficient of this peak (∼1900 M^–1^ cm^–1^) was considerably lower than what is typically reported for laccases (4000–6000 M^–1^ cm^–1^),^57,58^ again pointing to an atypical configuration at the T1 site. This reduced intensity suggests a less covalent Cu–S (Cys) bond (Cys329 in Figure 1A) or a perturbed T1 site geometry, possibly due to stronger axial methionine coordination or other second-sphere residue effects. Similar observations have been reported in nitrite reductases, which also has a T1 site, where a shorter Cu–S­(Met) bond was shown to weaken the Cu–S­(Cys) interaction and alter the geometry of the T1 site.^59^

In addition to the T1 feature, a distinct absorption at ∼330 nm was present, attributable to the oxidized T3 copper pair (the T2 copper is UV–vis silent). Upon addition of excess hydroquinone, both the 600 and 330 nm peaks decreased in intensity within 10 s (Figure 2B). This spectral behavior aligns with previous studies of MCOs where reduction leads to a loss of these absorbance features due to reduction of all copper sites.^60,61^ Kinetic traces of the decay of the 600 nm signal could not be adequately fit by a single exponential function, as also observed for other MCOs.^61^ Instead, the data was best described by a biexponential model, indicative of at least two distinct kinetic phases (Figure 2A, inset). The faster phase had a decay rate (i.e., rate of reduction) of 2.70 ± 0.05 s^–1^, while the rate for the slower phase was 0.37 ± 0.03 s^–1^. These findings are consistent with relatively fast initial reduction of the T1 copper site followed by intramolecular electron transfer to the TNC, with the slower phase representing a rate-limiting step in the electron transfer from T1 to TNC. Slow reduction of the RO state has been observed for high-turnover fungal laccases, such as that from *Rhus vernicifera* (*Rv*Lac), which nonetheless exhibits turnover rates of up to 560 s^–1^.^62^ In such efficient enzymes, catalysis proceeds through a native intermediate (NI), an oxidized state distinct from the resting oxidized enzyme. An enzyme in the NI state is reduced far more rapidly than the RO.^56,63^ Reduction of the RO state is inherently slow (∼1 s^–1^ for *Rv*Lac) and is not catalytically relevant under steady-state conditions for high-turnover laccases. In contrast, the steady-state turnover rate determined for *Cj*MCO (see below) closely matched the slow rate (0.37 s^–1^) for reduction of the RO, suggesting that the catalytically relevant oxidized state for *Cj*MCO might be the RO state. This behavior resembles the yeast ferroxidase Fet3p, where reliance on the RO state was shown to underlie its ∼10^3^-fold slower turnover relative to *Rv*Lac.^61^

Mixing the fully reduced (i.e., reduction of all four coppers) *Cj*MCO with O_2_ resulted in recovery of the absorption at 600 nm, corresponding to reoxidation of the T1 copper center (Figure 2C and D). The kinetics of this reoxidation process were remarkably slow, with complete reoxidation occurring on a seconds time scale at 25 °C. A biexponential fit yielded a fast initial phase with a rate of 3.1 ± 0.2 s^–1^ followed by a slower phase with a rate of 0.33 ± 0.1 s^–1^. This is in contrast to well-characterized MCOs such as the *Trametes versicolor* laccase, where reoxidation of the T1-copper occurred with observed rate constants >500 s^–1^ at a temperature of 4 °C.^60,61^ We propose that this slow reoxidation reflects constraints imposed by the quaternary structure, in which the TNC sits at the interface between protomers (Figure 1A), limiting efficiency of reoxidation compared with monomeric laccases.

Taken together, the correspondence between the steady-state turnover number (see below), the slower phase of RO reduction, and the rate of reoxidation suggests two possibilities for the limiting factor of the turnover number of *Cj*MCO. First, the RO state may represent the catalytically relevant oxidized form in the catalytic cycle, rather than an NI, although this remains uncertain. Second, and more definitively, overall turnover cannot exceed the rate of reoxidation by O_2_. This indicates that slow reoxidation by O_2_ imposes an inherent ceiling on the turnover number. Very few studies have measured reoxidation rates in bacterial laccases. Our results suggest that slow reoxidation by O_2_ could be a previously unrecognized factor contributing to the lower turnover number of bacterial laccases compared with fungal laccases.

### Catalytic Parameters Are Affected by Buffer Ions, Ionic Strength, and pH

To investigate how the unique structural and redox features of *Cj*MCO influence catalysis, we measured its activity toward different common oxidoreductase substrates (Figure 3). Oxidation of ABTS yielded a *k*_*cat*_ of 0.35 s^–1^ (Figure 3A), matching the slower phase of reduction by hydroquinone and reoxidation observed in the stopped-flow experiments (Figure 2). The catalytic activity of *Cj*MCO was inhibited at higher ABTS concentrations. Additionally, *Cj*MCO demonstrated metallooxidase activity, oxidizing Fe­(II) to Fe­(III) with a *k*_*cat*_ of 0.43 s^–1^ (Figure 3B). Of note, *Cj*MCO^as is^ (not copper saturated), had negligible activity (Figure S6), as copper reconstitution was needed for *Cj*MCO to bind copper in the T1 site (Figure S5). Truncation of the flexible His-region in *Cj*MCOΔ25–66 did not alter the apparent turnover number for ABTS oxidation compared to the wild type under identical conditions (Figure S6). For 2,6-DMP, the apparent turnover number was higher. This suggests that, at least for these substrates at pH 6.0, the flexible His-rich N-terminal region does not have a large effect on the enzyme’s catalytic efficiency. To examine *Cj*MCOs phenol oxidation kinetics, a key activity relevant to lignin degradation, we used 2,6-dimethoxyphenol (2,6-DMP) as a substrate.

**Figure 3:**
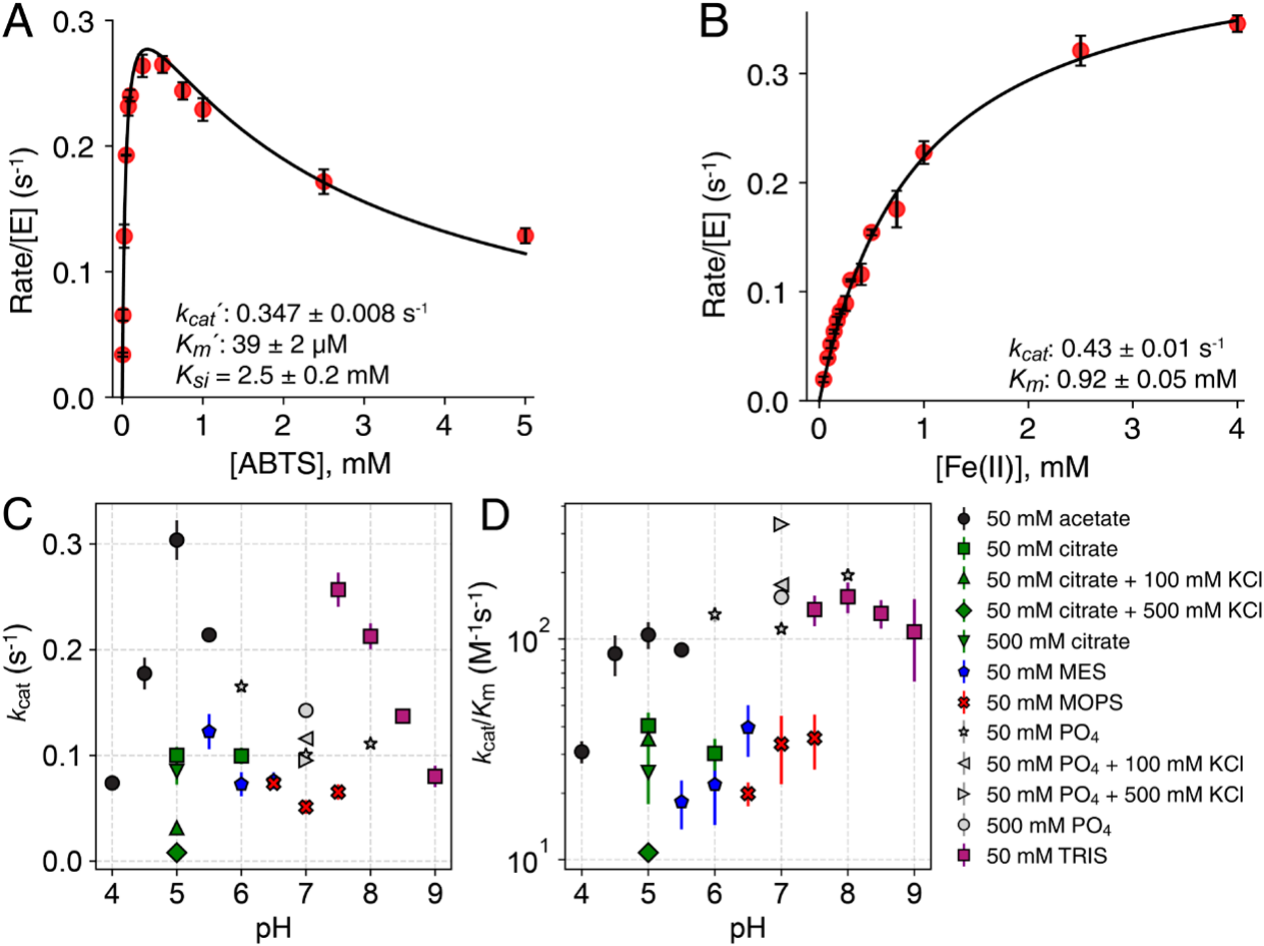
Kinetics of ABTS, Fe­(II), and 2,6-DMP oxidation by *Cj*MCO. (A) Oxidation kinetics for ABTS in 100 mM sodium acetate, pH 5.5, at 25 °C. Kinetic parameters were obtained by fitting (black line) data (red dots) to the substrate inhibition model: $v=\frac{V^{\prime }\times a}{\left(K_{m}^{\prime }+a+a^{2}/K_{\mathrm{si}}\right)}$ where *v* is the initial velocity, *V*′ is the apparent maximum reaction rate, K_m_′ is the apparent Michaelis–Menten constant in the presence of substrate inhibition, *a* is the substrate concentration, and *K_si_* is the substrate inhibition constant. (B) Oxidation of Fe­(II). The kinetic parameters were obtained by fitting (black line) the experimental data (red dots) to the Michaelis–Menten equation. (C) *k_cat_* and (D) *k_cat_/K_m_* values for 2,6-DMP oxidation under varying pH and buffer conditions as indicated in the legend on the right-hand side of panel D. For some data points, the standard deviations are smaller than the symbol size and are not visible. See Table S2 for a complete list of *K_m_*, *k_cat_*, and *K_m_*/*k_cat_* values.

The specific activity of *Cj*MCO toward 2,6-DMP in 50 mM sodium acetate buffer at pH 5.0 was 0.28 ± 0.02 μmol min^–1^ mg^–1^. Michaelis–Menten kinetic analysis of 2,6-DMP oxidation by *Cj*MCO further revealed complex dependencies, with catalytic parameters being affected not only by pH but also, and sometimes more strongly, by buffer composition and ionic strength (Figure 3C and D, Table S2). For instance, at pH 5.0, switching from sodium acetate to citrate buffer reduced *k*_*cat*_ and slightly decreased *K*_*m*_. A similar trend was observed at pH 7.0, where replacing MOPS with sodium phosphate doubled *k*_*cat*_ and lowered *K*_*m*_. Moreover, pH and ionic strength showed nuanced interplay. In sodium phosphate buffer at pH 7.0, increasing ionic strength with KCl enhanced catalytic efficiency by lowering *K*_*m*_, despite a modest decrease in *k*_*cat*_. In contrast, in citrate buffer at pH 5.0, increasing ionic strength reduced *k*_*cat*_, and at 500 mM KCl, enzymatic activity was nearly abolished. These effects highlight the role of electrostatic interactions in substrate binding and catalysis. Of note, next to effects on enzyme–substrate interactions and the properties of catalytically important residues, the observed effects are likely to also relate to direct interactions between ionic compounds in the solution and one or more of the bound copper ions, e.g., for citrate.^64^ Such effects have recently been described for copper-dependent lytic polysaccharide monooxygenases.^65,66^ Overall, these findings underscore that the activity of *Cj*MCO is tuned by its local chemical environment. Given the condition-dependent behavior, it is impractical to define a single optimal pH for *Cj*MCO. Instead, its activity spanned a broad pH range, with buffer system and ionic strength acting as factors modulating catalytic efficiency. Importantly, while reported turnover frequencies for bacterial enzymes on 2,6-DMP typically range from ∼0.01 s^–1^ to ∼30 s^–1^,^67–69^ most characterized bacterial laccases exhibit turnover frequencies above 1 s^–1^. In this context, the turnover frequency of *Cj*MCO, which was below 1 s^–1^, is at the lower end of this spectrum.

### *Cj*MCO Oxidized, Polymerized and Cleaved a β-*O*-4 Lignin Model Dimer

We next investigated whether *Cj*MCO’s substrate tunnel allowed oxidation of larger substrates, using dimeric β-*O*-4 lignin model compounds. Like all laccases, *Cj*MCO had a lower reduction potential than lignin peroxidases, which limits its ability to oxidize nonphenolic aromatic compounds. As expected, no activity was detected toward the nonphenolic model compounds veratryl alcohol or veratrylglycerol-β-guaiacyl ether (VBG) (data not shown). Of note, *Cj*MCO neither showed any detectable conversion of VBG when incubated with various mediators (Figure S7). However, *Cj*MCO did oxidize the phenolic model guaiacylglycerol-beta-guaiacyl ether (GBG) (Figure 4A), demonstrating its ability to accommodate larger substrates near the T1 active sites. Conversion (defined by substrate disappearance) was more efficient at higher pH than at lower pH (Figure 4B), in contrast to many fungal laccases which tend to show optimal activity at acidic pH.

**Figure 4:**
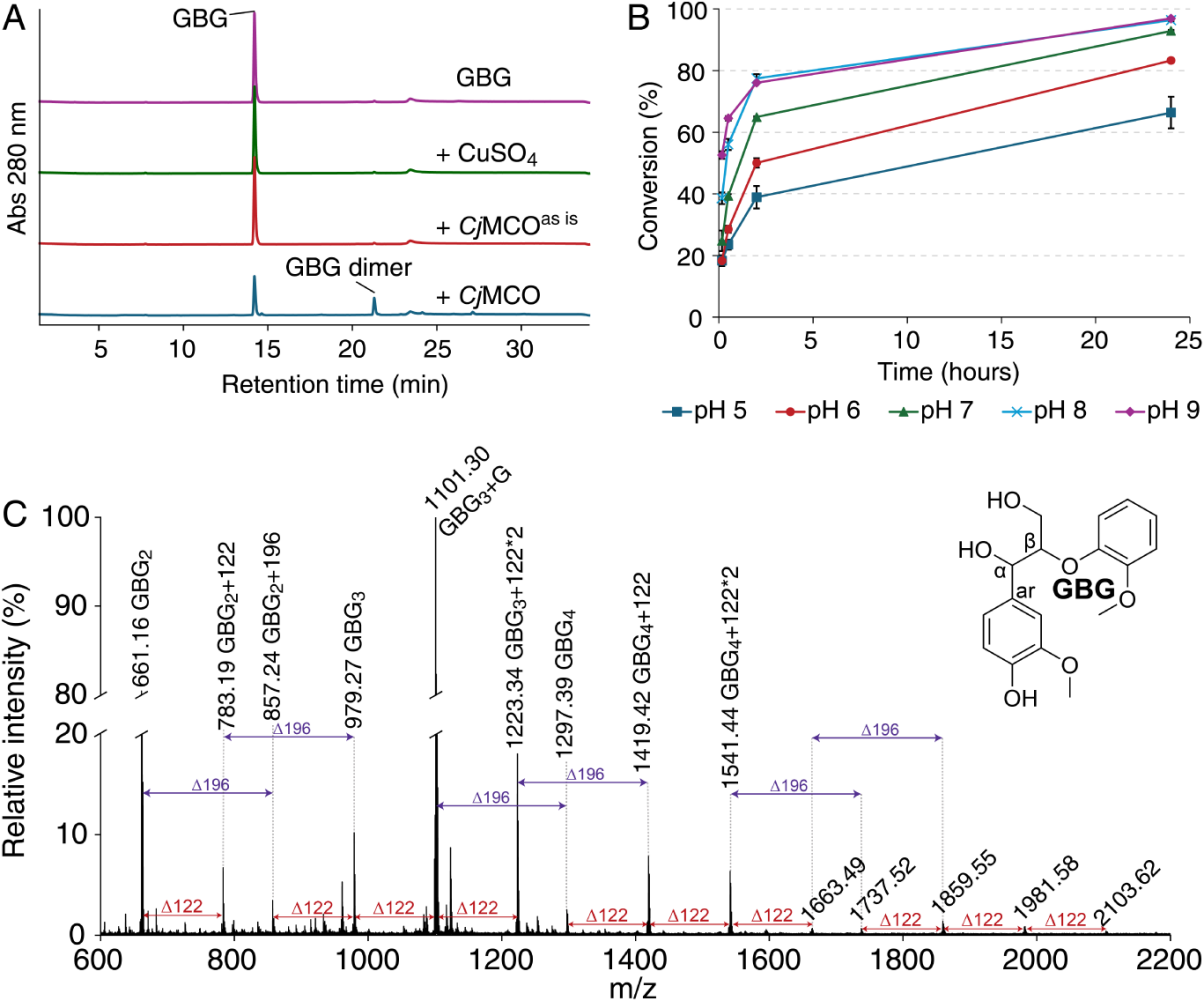
GBG oxidation by *Cj*MCO. **(A)** UHPLC profiles showing oxidation of GBG by *Cj*MCO. Reactions were performed in 50 mM sodium phosphate, pH 7.0, at 40 °C with 200 μM GBG and 1 μM *Cj*MCO (blue line) or *Cj*MCO^as is^ (red line). In the control reaction with CuSO_4_ (green line), CuSO_4_ was added to a final concentration of 4 μM. The reactions were incubated for 2 h. (B) Conversion of GBG over time at different pHs. Reactions containing 200 μM GBG and 1 μM *Cj*MCO were performed at 30 °C in a combined buffer (20 mM sodium acetate, 20 mM sodium phosphate, and 20 mM Tris-HCl) at different pHs. Error bars show standard deviation (*n* = 3). (C) MALDI-TOF MS analysis of oligomeric compounds formed by *Cj*MCO after 24 h incubation with GBG. All species labeled with their *m*/*z* values are [M + Na]^+^ species. Red and purple arrows represent mass shifts of +122 Da or +196 Da. Table S4 provides more details for selected oligomeric compounds and their assignment. Figure S9 shows the structures of possible products.

GBG oxidation by *Cj*MCO primarily yielded higher-molecular-weight oligomers, formed through radical coupling (Figure 4C). UHPLC-MS analysis (Figure S8) confirmed the presence of the GBG dimers visible in Figure 4A, with retention time and fragmentation matching data published previously.^15^ The analysis also revealed several additional products not attributable to simple polymerization of GBG (Figures S8 and S9, Tables S3 and S4). One such product is Cα-oxidized GBG (*m*/*z* 318), again with matching retention time and fragmentation. Other products, to the best of our knowledge previously unpublished, were detected at *m*/*z* 471 and *m*/*z* 759, respectively, matching a *net* increase of 152 Da relative to GBG and *net* increase of 122 Da relative to a GBG dimer.

The MS^2^ fragmentation behavior of the *m*/*z* 471 product was identical to GBG, with all fragments being increased with the 152 Da as observed for the parent ion (Figure S9). The *m*/*z* 439 and 423 fragments implied that the GBG aryl ether structure remained unchanged, while the *m*/*z* 347 fragment indicated that the 152 Da was added to the phenolic ring. The absence of other diagnostic fragments hampered further identification of the coupled structure. Given the guaiacyl nature of the substrate, we consider it most likely that the coupled structure is guaiacylic as well, from which vanillyl alcohol followed as tentatively coupled product (Figure S9). The *m*/*z* 759 product likewise did not show diagnostic fragments (Figure S9). Following the same line of thought for the *m*/*z* 471 product, the *net* increase of 122 Da most likely originated from the *net* oxidative coupling of guaiacol (C_7_H_8_O_2_, 124.1 Da).

To further characterize these coupled oligomeric products, and especially those of higher molecular weight, MALDI-TOF MS analysis was performed (Figure 4C, Table S4). As anticipated, we detected dimers, trimers, and tetramers of GBG. However, similar to the UHPLC-MS results, several ions could not be attributed to simple oxidative oligomerization of GBG. The most prominent peak at *m*/*z* = 1101 was attributed to a GBG trimer +122 Da. Further prominent ions, such as those at *m*/*z* = 783 and *m*/*z* = 857, exhibited net mass increases of 122 and 196 Da relative to a GBG dimer at *m*/*z* = 661. No products with a *net* increase of 152 Da were observed.

In fact, two series of compounds with respective *m*/*z* shifts of +122 Da and +196 Da were observed. We preliminary attribute the +122 Da series to the *net* addition of guaiacol to GBG oligomers, but currently do not understand the origin of all +196 Da structures observed (e.g., *m*/*z* 857). High-resolution mass spectrometric analysis to give insight into the elemental composition and/or more advanced MS^*n*^ fragmentation analysis could help to unambiguously assign the structures formed.

Though the exact products remained unidentified, they must be the result of *net* cleavage and either formed through coupling of initially cleaved structures, through cleavage of initially coupled structures or even through cleavage as a result of coupling. Speculating on the exact structures and underlying reaction mechanisms goes beyond the scope of the current work. Collectively, however, these results indicated that GBG oxidation by *Cj*MCO induced not only the radical-mediated polymerization of GBG but also facilitated bond cleavage. While these cleavage products were admittedly detected at low levels compared to GBG dimers only, their occurrence is noteworthy as they have not (to our knowledge) previously been reported for GBG oxidation by laccases.

The reaction pathways leading to either GBG-coupling or cleavage after one-electron oxidation are strictly nonenzymatic in nature and should therefore not be unique to *Cj*MCO. Expectedly, using *Trametes versicolor* laccase under identical conditions (i.e., pH 7.0) yielded a similar product profile (Figure S10), indicating that such mixed reactivity could be a general feature of laccase activity on phenolic β-*O*-4 lignin dimers. Of note, *Tv*Lac is typically employed at acidic pH. Since the reaction pathways that resulted in β-*O*-4 cleavage likely proceeded via nonenzymatic radical intermediates, the balance between coupling and cleavage likely depends on pH, buffer composition, and other reaction conditions. Figure S11 shows that the pH and ionic strength indeed influenced product formation in these reactions, providing a way of controlling the reaction outcome. A previous study has shown that buffer strength and pH affect product profiles generated by laccase mediator systems acting on (nonphenolic) veratrylglycerol-β-guaiacyl ether.^70^ We show here how reaction conditions also shaped product profiles for oxidation of phenolic structures. While a more comprehensive study is needed to fully elucidate the underlying mechanisms, our results suggest that optimization of conditions can tune both enzyme efficiency and product profile.

### *Cj*MCO Oxidized Oligomeric Organosolv Lignin

Since *Cj*MCO was able to oxidize GBG, and caused both polymerization and bond cleavage, we next examined its activity on a larger lignin substrate, obtained from birch wood through organosolv processing. The organosolv lignin used for these reactions was of relatively low molecular weight (*M*_*w*_ average 1650 g/mol, Figure 5A), had high S/G (3.5, Table S6), was relatively high in β-*O*-4 aryl ether content (25 per 100 aromatic units, Table S6), and contained 1.2 mmol/g phenolic groups (Table S5). The presence of phenolic groups makes it a suitable substrate to evaluate *Cj*MCO activity. Despite the potentially limited access to the active site suggested previously, *Cj*MCO was able to act on this oligomeric lignin. The activity of *Cj*MCO on organosolv lignin led to a net polymerization, shifting its average molecular weight from 1650 g/mol to 6500 g/mol (Figure 5A), exceeding the minor increase in molecular weight observed for control reactions (to 2800 g/mol for *Cj*MCO^as is^ and 2020 g/mol for CuSO_4_).

**Figure 5:**
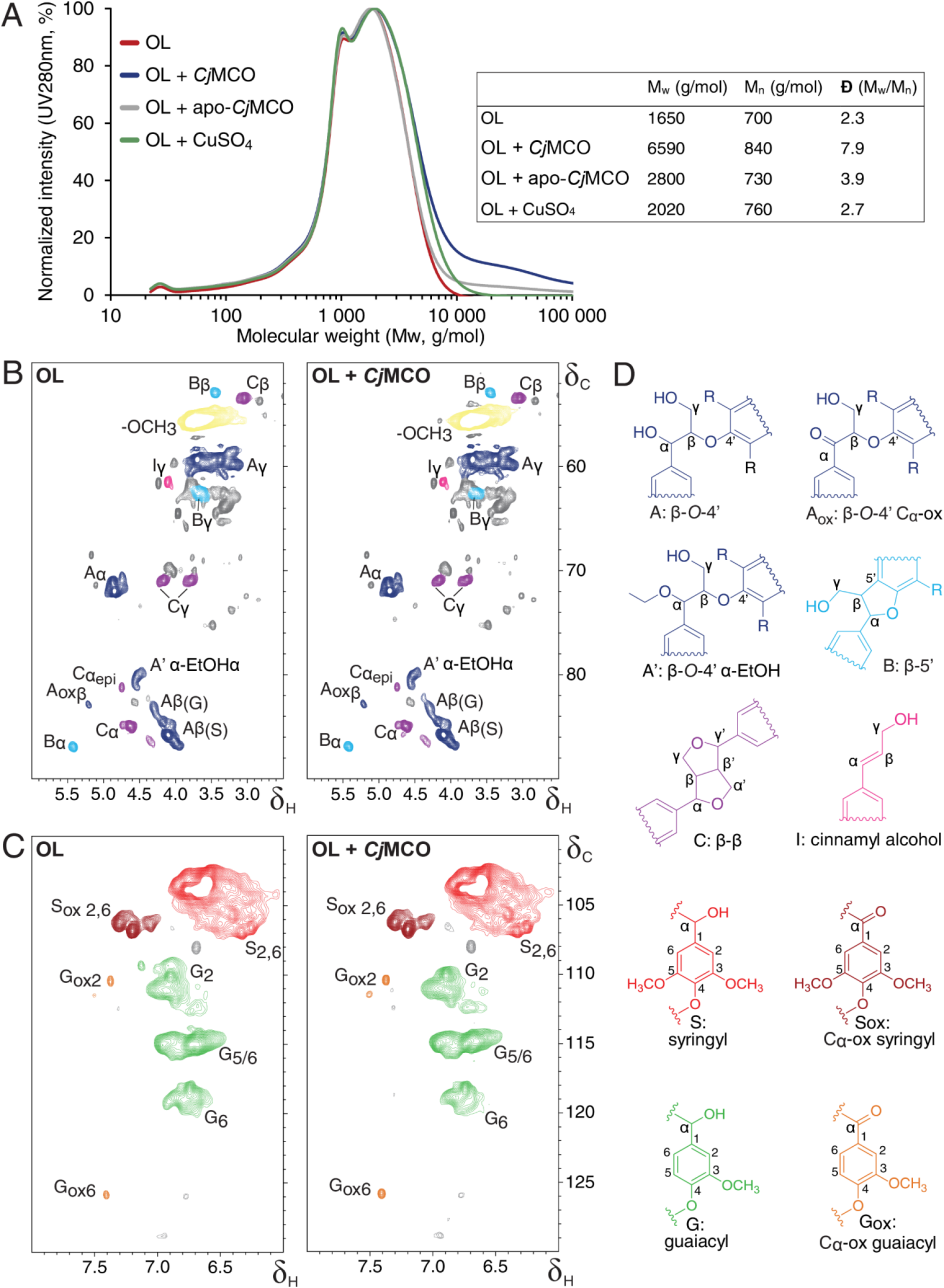
Incubation of *Cj*MCO with organosolv lignin (OL) from birch analyzed by 2D-HSQC NMR and SEC. All incubations were performed in 25% (v/v) acetonitrile, 10 mM sodium phosphate, pH 7.0, in 50 mL tubes that were incubated at 25 °C for 18 h with shaking at 150 rpm. Reactions with enzyme were started by adding *Cj*MCO or *Cj*MCO^as is^ to a final concentration of 5 μM. (A) SEC profiles of OL (red line), and of OL incubated with *Cj*MCO (blue line), *Cj*MCO^as is^ (gray line), or 30 μM CuSO_4_ (green line). The chromatogramss show the relative UV absorbance at 280 nm, normalized to the maximum intensity observed in each reaction. Inset table shows: *M*_*w*_: Weight-average molecular weight, *M*_*n*_: Number-average molecular weight, Đ: Polydispersity index (the ratio of *M*_*w*_ to *M*_*n*_). (B) Aliphatic and (C) aromatic regions of the 2D-HSQC NMR spectra. See Figure S12 for spectra of control reactions. Greek letters and numbers indicate the specific carbon/hydrogen positions giving rise to each signal in substructures shown in panel D. “epi” refers to the epimer of the beta–beta resinol structure. (D) Annotated lignin substructures, with colors corresponding to signals in panels B and C. “R” denotes either hydrogen or a methoxy group. Unassigned peaks are shown in gray.

To assess potential structural changes in the residual lignin beyond polymerization, 2D-HSQC NMR spectroscopy was performed. HSQC spectra of *Cj*MCO-treated organosolv lignin showed strong overlap with control spectra, indicating that the lignin substructures remained largely intact (Figure 5B–D, Figure S12, Table S6). Semiquantitative analysis showed a minor increase in C_α_-oxidation signals and an increased syringyl/guaiacyl ratio, but similar changes were also present in CuSO_4_-treated controls. It must be noted that the latter reaction contained a high concentration of “free” copper, potentially underlying the slight oxidation observed, but without leading to severe polymerization as inferred from SEC (Figure 5A).

A word of caution is added to the interpretation of the HSQC NMR spectra and corresponding semiquantitative analysis here. HSQC NMR relies on the detection of protons that are coupled to carbons. Therefore, the absolute intensity of the G_2_, G_5_, G_6_ and S_2,6_ signals reflects the entire detectable aromatic lignin subunit population. The resolved G_2_ and S_2,6_ are typically used for semiquantitative purposes, where their sum is put at 100% and all other signals are expressed relative to this number, also referred to as “structural moiety per 100 aromatic rings” (Table S6). Care should be taken when lignin condenses, as this leads to a decrease in detectable C–H bonds while the actual number of aromatic subunits per gram of lignin remains constant. Since guaiacyl units are most prone to condense, we compared the volume integral of the G_2_ signal to that of the well-resolved methoxyls of which the detection is not impacted by condensation (Table S6). Indeed, the G_2_/methoxyl ratio decreased most in the *Cj*MCO treated lignin, implying that this lignin is most condensed and fully aligned with SEC analysis.

Since interunit linkages and end-units are quantified relative to G_2_ and S_2,6_ signals, their apparent increase (Table S6) is most likely the result of this condensation and should rather be considered an analytical artifact of HSQC NMR, rather than reflecting true structural changes. Together, the SEC and HSQC NMR results show that under the tested conditions, *Cj*MCO primarily acts on oligomeric organosolv lignin via oxidative coupling, with minimal bond cleavage.

The finding that *Cj*MCO is able to act on large­(r) lignin structures prompts the question how substrate accessibility is organized in the unique structural framework of the protein with its active site hidden in a tunnel, also in relation to reaction environment in terms of buffer ions, ionic strength and pH. Based on our observations that these parameters influence catalytic properties, they might provide handles for future finetuning of the reaction outcome.

## Conclusion

In this study, we have characterized a MCO from *Cellvibrio japonicus*, a member of the structurally distinct and relatively unknown type B 2dMCOs. *Cj*MCO featured a trimeric architecture, with T1 copper sites oriented toward a central tunnel and an interfacial TNC. *Cj*MCO functioned as a laccase, evident by its activity and substrate promiscuity. However, its turnover frequency was ∼1000 fold slower than high-turnover fungal laccases. We propose that *Cj*MCO’s turnover is inherently limited by the slow rate of O_2_ reoxidation. Nevertheless, *Cj*MCO displayed broad substrate specificity, oxidizing common oxidoreductase substrates, Fe^2+^, and the lignin β-*O*-4 dimeric model compound GBG. Interestingly, the analysis of the GBG reactions revealed a wide range of products resulting from oxidative coupling as well as cleavage. Ionic strength and pH affected product profiles from GBG oxidation, providing a possible tool to control the products generated. The enzyme was further able to act on oligomeric lignin, again showing oxidative polymerization. Our results therefore imply that *Cj*MCO could contribute to lignin modification or degradation under biological conditions. However, the slow reaction rates and the large trimeric structure suggest that *Cj*MCO is more likely specialized for roles other than the efficient oxidation of large lignin polymers *in vivo*. Thus, while the involvement of *Cj*MCO in lignin degradation cannot be ruled out, its principal physiological role may lie elsewhere. Plausible alternatives include detoxification of phenolic compounds, copper binding or metal homeostasis. The slow O_2_-dependent reoxidation kinetics could indicate adaptation to low-oxygen environments, or that O_2_ is not its preferred physiological oxidant. Overall, this work expands our understanding of bacterial MCOs diversity and provides a foundation for future investigations into the biological functions of 2dMCOs.

## Supplementary Material



## Notes

Multiple sequence alignment and AlphaFold structures of *Cellvibrio* multicopper oxidases; native PAGE analysis of *Cj*MCO; *Cj*MCO^as is^; *Cj*MCOΔ25–66; *Cj*MCOΔ25–66^as is^; AlphaFold-generated homotrimeric structure of *Cj*MCO with bound copper; UV–vis spectra of *Cj*MCO and *Cj*MCO^as is^; ABTS and DMP oxidation by *Cj*MCO and *Cj*MCOΔ25–66; UHPLC profiles of mediators and veratrylglycerol-β-guaiacyl ether incubated with *Cj*MCO; UHPLC-MS analysis and proposed product structures from GBG oxidation by *Cj*MCO; MALDI-TOF MS spectra and oligomer identification of GBG oxidation products formed by *Cj*MCO and *Tv*Lac; UHPLC profiles of GBG oxidation across pH and ionic strength conditions; HSQC NMR spectra of organosolv lignin treated with *Cj*MCO or controls; kinetic parameters for DMP oxidation under varying buffer, pH, and ionic strength; compounds detected by UHPLC-MS after oxidation of GBG with *Cj*MCO; MALDI-TOF MS identification of oligomeric GBG oxidation products; hydroxyl content of birch organosolv lignin as determined by ^31^P NMR; NMR-derived structural parameters for lignin incubated with CjMCO or controls (PDF)

## Abbreviations

2dMCO: Two-domain multicopper oxidase
3dMCO: Three-domain multicopper oxidase
6dMCO: Six-domain multicopper oxidase
ABTS: 2,2′-azino-bis­(3-ethylbenzothiazoline-6-sulfonic acid)
*Cj*MCO: Type B 2dMCO from *Cellvibrio japonicus*
*Cj*MCOΔ25–66: Truncated variant of *Cj*MCO
2,6-DMP: 2,6-Dimethoxyphenol
GBG: Guaiacylglycerol-beta-guaiacyl ether
HBT: 1-hydroxybenzotriazole
OL: organosolv lignin
UHPLC-MS: Ultrahigh performance liquid chromatography–mass spectrometry
SEC: Size-exclusion chromatography
T1 copper site: Type-1 copper site
TNC: Trinuclear cluster
*Tv*Lac: Laccase from *Trametes versicolor*
