# Supplementary material for: Understanding Lignin Oxidation by a Two-Domain Multicopper Oxidase from Cellvibrio japonicus
Source: Biochemistry. 2026 Apr 27;65(9):1599–613. doi: 10.1021/acs.biochem.6c00183 (PMC13151041; doi:10.1021/acs.biochem.6c00183)
Supplement: Supplementary file 1 [file bi6c00183_si_001.pdf]

Supporting information

Understanding lignin oxidation by a two-domain multicopper oxidase from *Cellvibrio japonicus*

Morten Rese[1], Gijs van Erven[2,3], Mirjam A. Kabel[3], Anton A. Stepnov[1] , Vincent G. H. Eijsink[1], Tina R. Tuveng[1].*

[1]Faculty of Chemistry, Biotechnology, and Food Science, Norwegian University of Life Sciences (NMBU), P.O. Box 5003, 1433 Ås, Norway

[2]Wageningen Food and Biobased Research, Bornse Weilanden 9, 6708 WG, Wageningen, The Netherlands

[3]Wageningen University & Research, Laboratory of Food Chemistry, Bornse Weilanden 9, 6708 WG, Wageningen, The Netherlands

*Corresponding author (Email: tina.tuveng@nmbu.no)

Contents

Figure S1. Multiple sequence alignment and AlphaFold structures of 14 multicopper oxidases from the *Cellvibrio* genus.

Figure S2. Native PAGE of *Cj*MCO and *Cj*MCOΔ25–66.

Figure S3. AlphaFold structure of homotrimeric *Cj*MCO with copper atoms.

Figure S4. Determination of the reduction potential.

Figure S5. UV-Vis spectra of *Cj*MCO and *Cj*MCO[as is].

Figure S6. ABTS and DMP oxidation by *Cj*MCO and *Cj*MCOΔ25–66.

Figure S7. UHPLC profiles of various mediators and VBG incubated with *Cj*MCO.

Figure S8. UHPLC-MS analysis of products from GBG oxidation by *Cj*MCO.

Figure S9. Proposed product structures resulting from GBG oxidation by *Cj*MCO.

Figure S10. MALDI-ToF MS spectra of GBG oxidized by *Cj*MCO and *Tv*Lac.

Figure S11. HPLC profiles of GBG oxidation by *Cj*MCO under varying pH and ionic strength.

Figure S12. HSQC NMR spectra of organosolv lignin treated with *Cj*MCO or controls.

Table S1: Copper content quantification of *Cj*MCO and *Cj*MCOΔ25–66 with and without copper reconstitution.

Table S2. Kinetic parameters for DMP oxidation under different buffer, pH, and ionic strength conditions.

Table S3. Compounds detected by UHPLC–MS after oxidation of GBG with *Cj*MCO.

Table S4. MALDI-TOF MS identification of oligomeric GBG oxidation products.

Table S5. Hydroxyl content (mmol/g) of birch organosolv lignin as determined by ^{31}P NMR.

Table S6. NMR-derived structural parameters for lignin incubated with *Cj*MCO or controls.

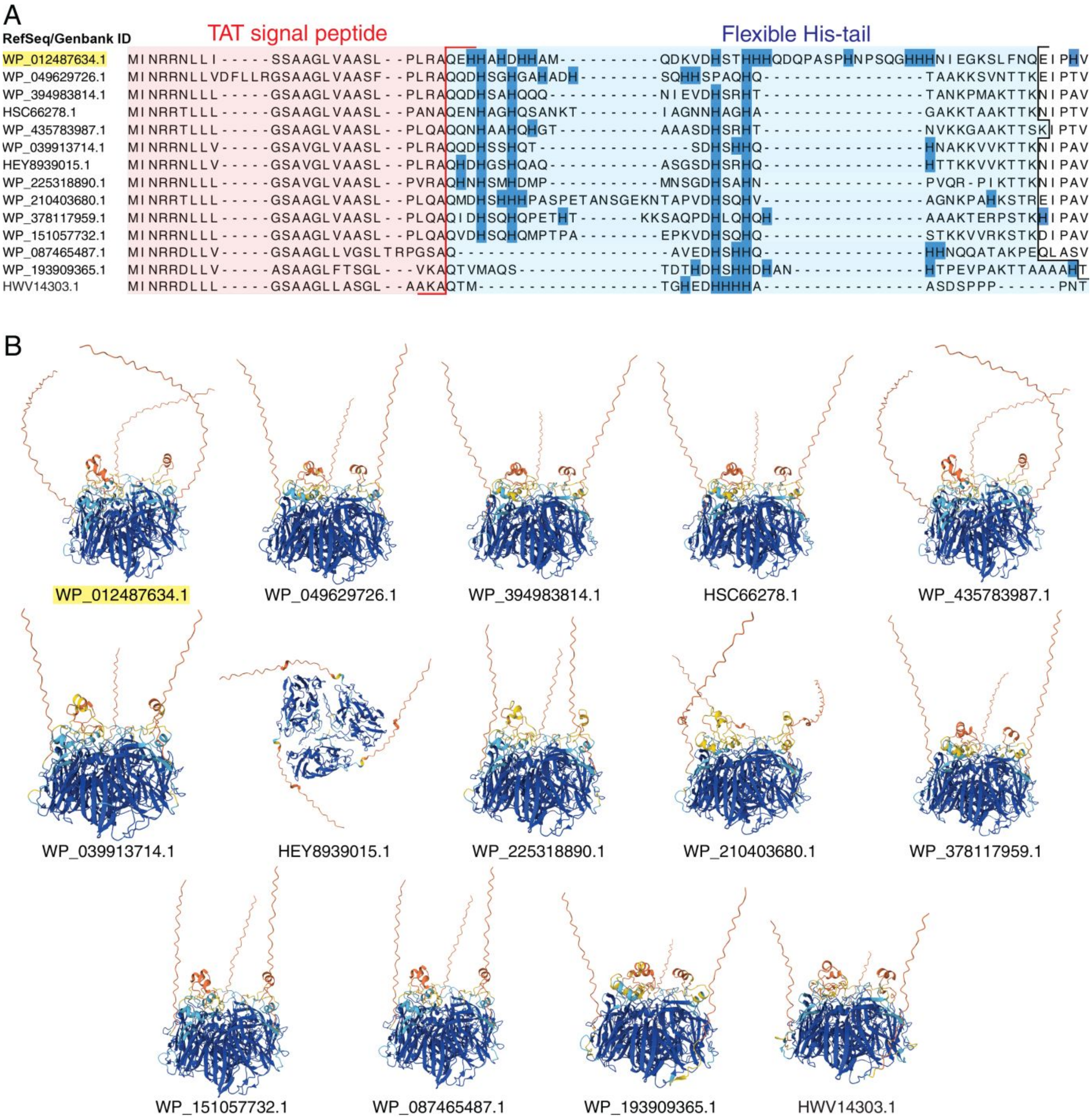

Figure S1. Multiple sequence alignment (MSA) and AlphaFold structures of 14 multicopper oxidases from the *Cellvibrio* genus, each harboring a flexible histidine-rich region. (A) MSA of the signal peptide and the N-terminal region generated using MAFFT with default parameters. RefSeq/GenBank accession numbers are provided for all sequences, with the multicopper oxidase from *Cellvibrio japonicus* (*Cj*MCO) highlighted in yellow. SignalP 6.0[1] was used to identify TAT signal peptides, indicated in red. The flexible histidine-rich region is marked in blue, based on regions assigned as structurally disordered with very low AlphaFold confidence (plDDT < 50). **(B)** AlphaFold structural predictions generated using three copies of the mature sequence and 12 copper ions as input. Structure confidence is colored: very high (plDDT > 90, dark blue); confident (90 > plDDT > 70, light blue); low (70 > plDDT > 50, yellow); and very low (plDDT < 50, orange). For clarity, structure HEY8939015.1 is shown rotated 90° relative to the others to better visualize its histidine-rich region.

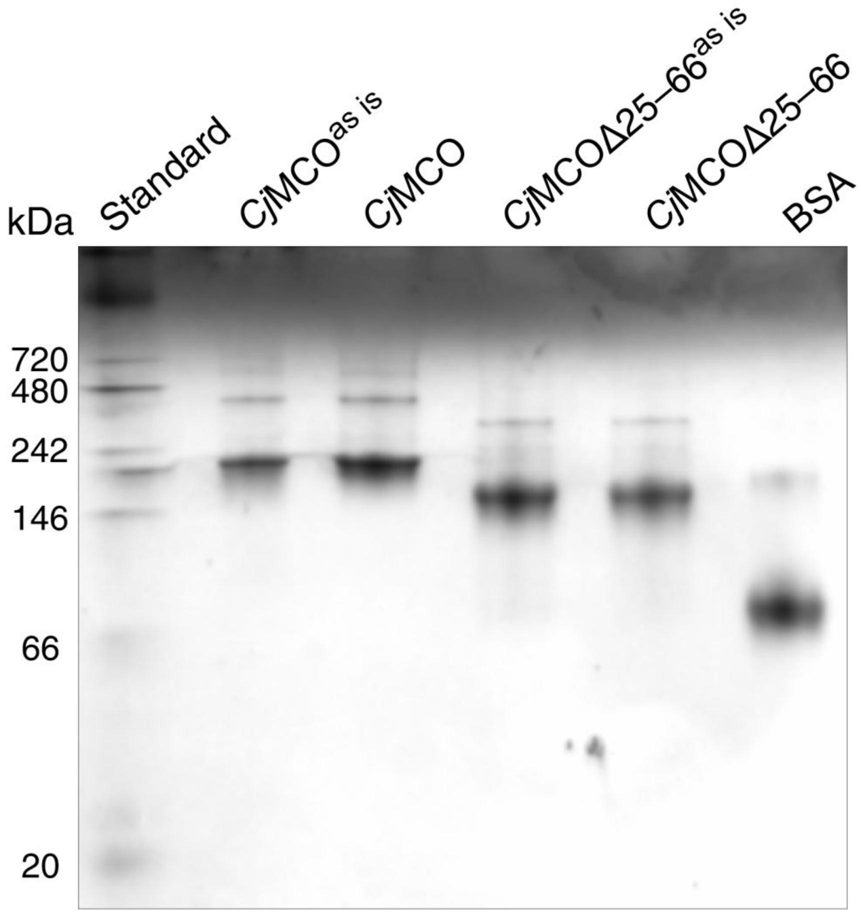

Figure S2. Native polyacrylamide gel electrophoresis of copper saturated, and non-copper saturated ("as is") *Cj*MCO and *Cj*MCOΔ25–66. Purified protein (4 μg) was applied in each well. Bovine serum albumin (BSA) with a theoretical molecular weight of 66 kDa was used as reference. The theoretical molecular weights of trimeric *Cj*MCO and *Cj*MCOΔ25–66 are 149 kDa and 134 kDa, respectively. NativeMark™ Unstained Protein Standard (Invitrogen) was used as standard. The gel was stained with Coomassie blue for visualization. The band appearing between 146 kDa and 242 kDa in the standard is spillover from the neighboring well.

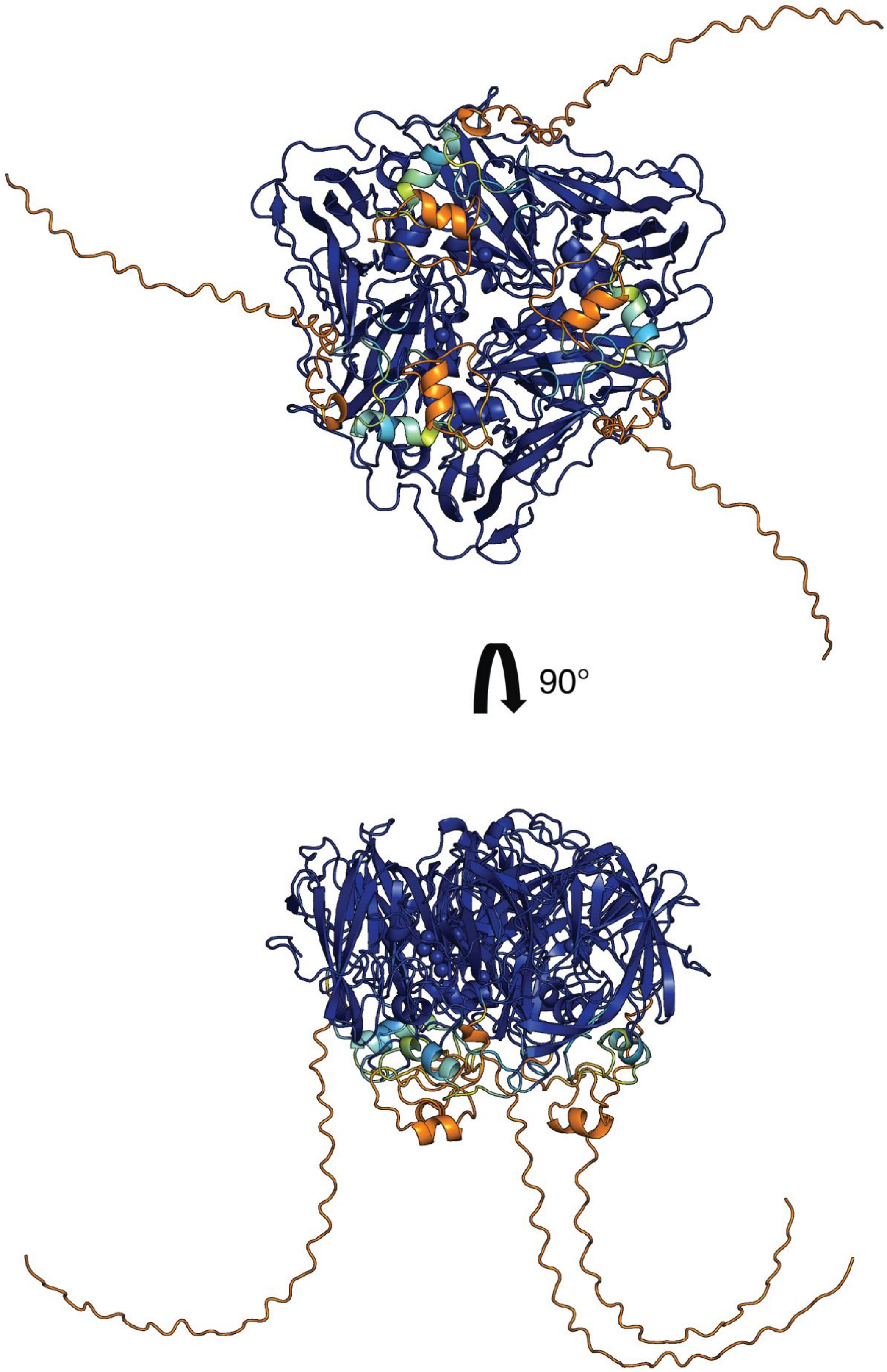

Figure S3. Alphafold structure of the homotrimeric Wt-*Cj*MCO complex shown as cartoon with copper atoms as spheres. Colors correspond to the plDDT score. Blue = Very high (plDDT > 90), Cyan = Confident (90 > plDDT > 70), Yellow = Low (70 > plDDT > 50), Orange = Very low (plDDT < 50). All Cu-atoms and all residues coordinating these Cu-atoms had a plDDT > 90.

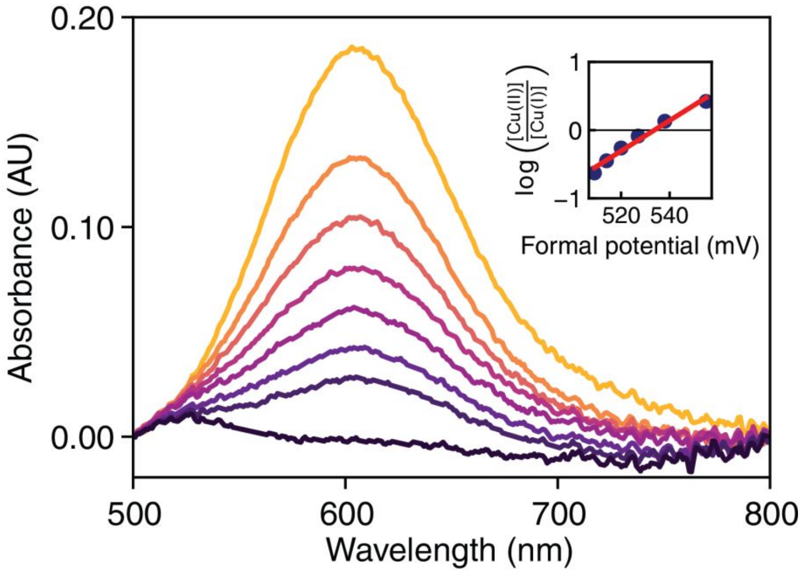

Figure S4. Determination of the reduction potential. The graphs show absorbance in the 500 – 800 nm region during poised potential titration of *Cj*MCO under anaerobic conditions using the $K_3[Fe(CN)_6]/K_4[Fe(CN)_6]$ redox couple. The inset shows the Nernst plot of log([Cu(II)]/[Cu(I)]) versus applied potential. An E° of 537 ± 7 mV was determined from three independent titrations. The fully oxidized and fully reduced spectra are shown in yellow and black, respectively. The Cu(II)/Cu(I) ratio is reflected in the absorbance at 600 nm during titration with $K_4[Fe(CN)_6]$.

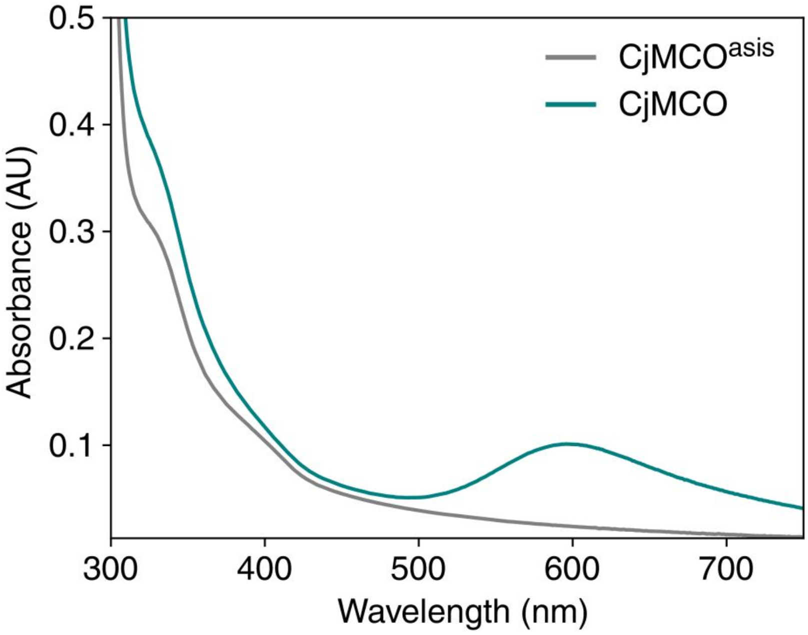

Figure S5: UV-Vis spectra of non-copper saturated *Cj*MCO (*Cj*MCO$^{as\ is}$, grey trace) and copper saturated *Cj*MCO (*Cj*MCO, teal trace). The spectra shows that *Cj*MCO exhibits a characteristic T1 Cu^{2+} absorption band near ~600 nm, whereas this feature is absent in *Cj*MCO$^{as\ is}$. Spectra were recorded in 50 mM MOPS buffer (pH 7.0) with a protein concentration of 60 μM.

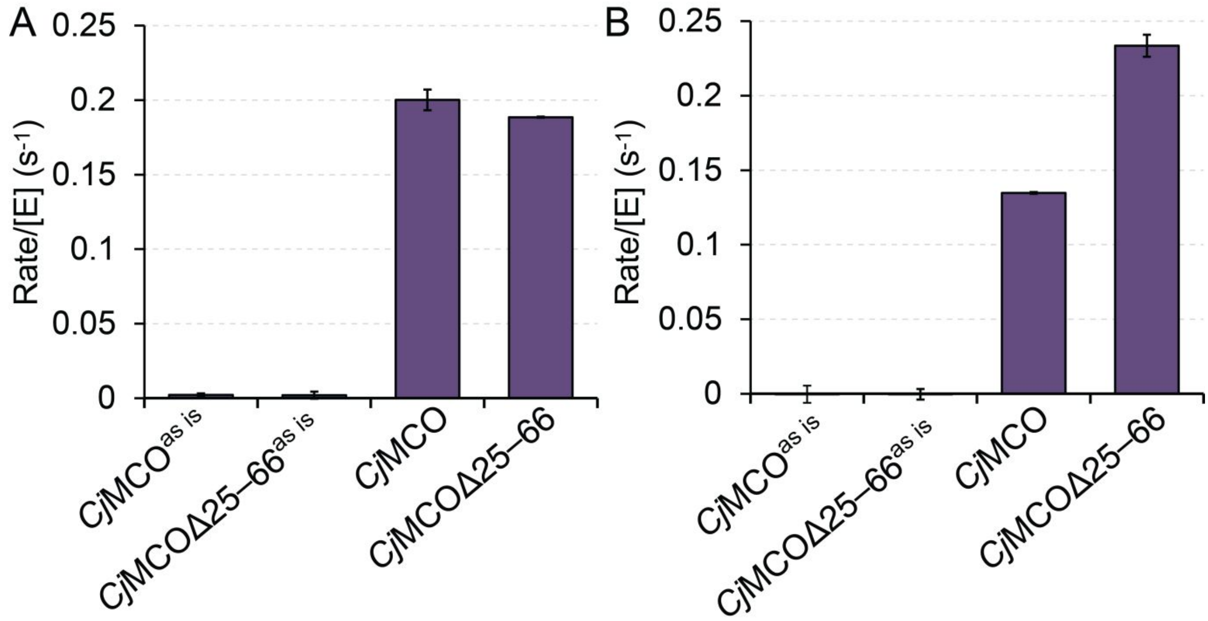

Figure S6. (A) ABTS and **(B)** 2,6-DMP oxidation by *Cj*MCO and *Cj*MCOΔ25–66. Reactions were performed in a 96-well plate with a total volume of 200 μL in 100 mM potassium phosphate (pH 6.0) at 25 °C. Concentration of ABTS and 2,6-DMP were 0.5 mM and 10 mM respectively. Initial rates divided by enzyme concentration are shown. Error bars show standard deviations (n=3).

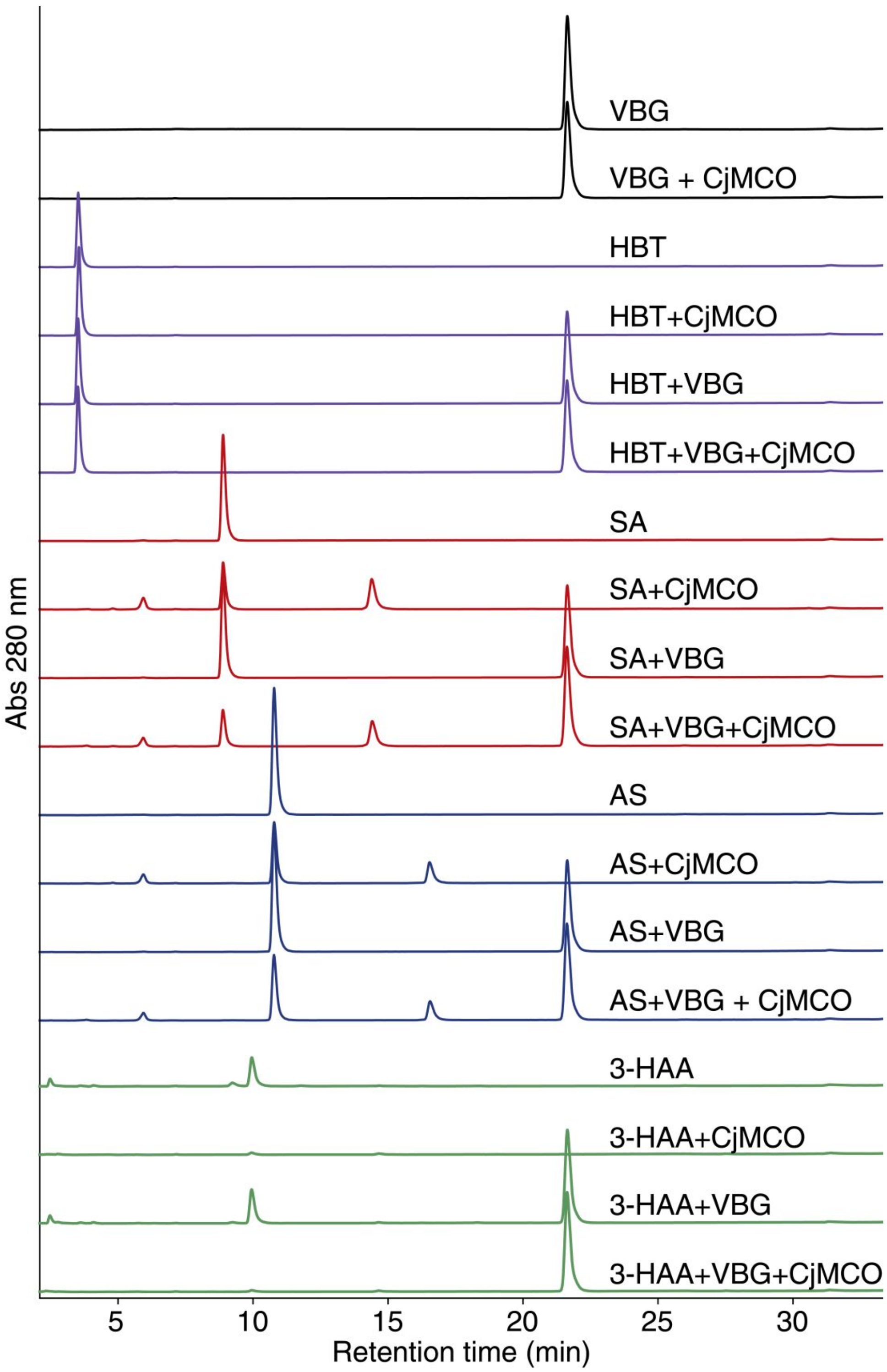

Figure S7. HPLC analysis of reactions with various *Cj*MCO-mediator combinations. The HPLC profile were obtained after incubating various compounds in the presence and absence of *Cj*MCO. Incubations included combinations of the nonphenolic lignin model dimer veratrylglycol-beta-guaiacyl ether (VBG), and the potential mediators hydroxybenzotriazole (HBT), syringic acid (SA), acetosyringone (AS), and 3-hydroxyanthranilic acid (3-HAA) at concentrations of 200 μM for VBG and 100 μM for mediators. The chromatograms show that VBG is not modified in any of the reactions,

while *Cj*MCO does react with two of the mediators, AS and SA. Reactions were carried out in 50 mM sodium phosphate buffer, pH 7.0, 1000 rpm, and 40 °C. The *Cj*MCO concentration was 1 μM.

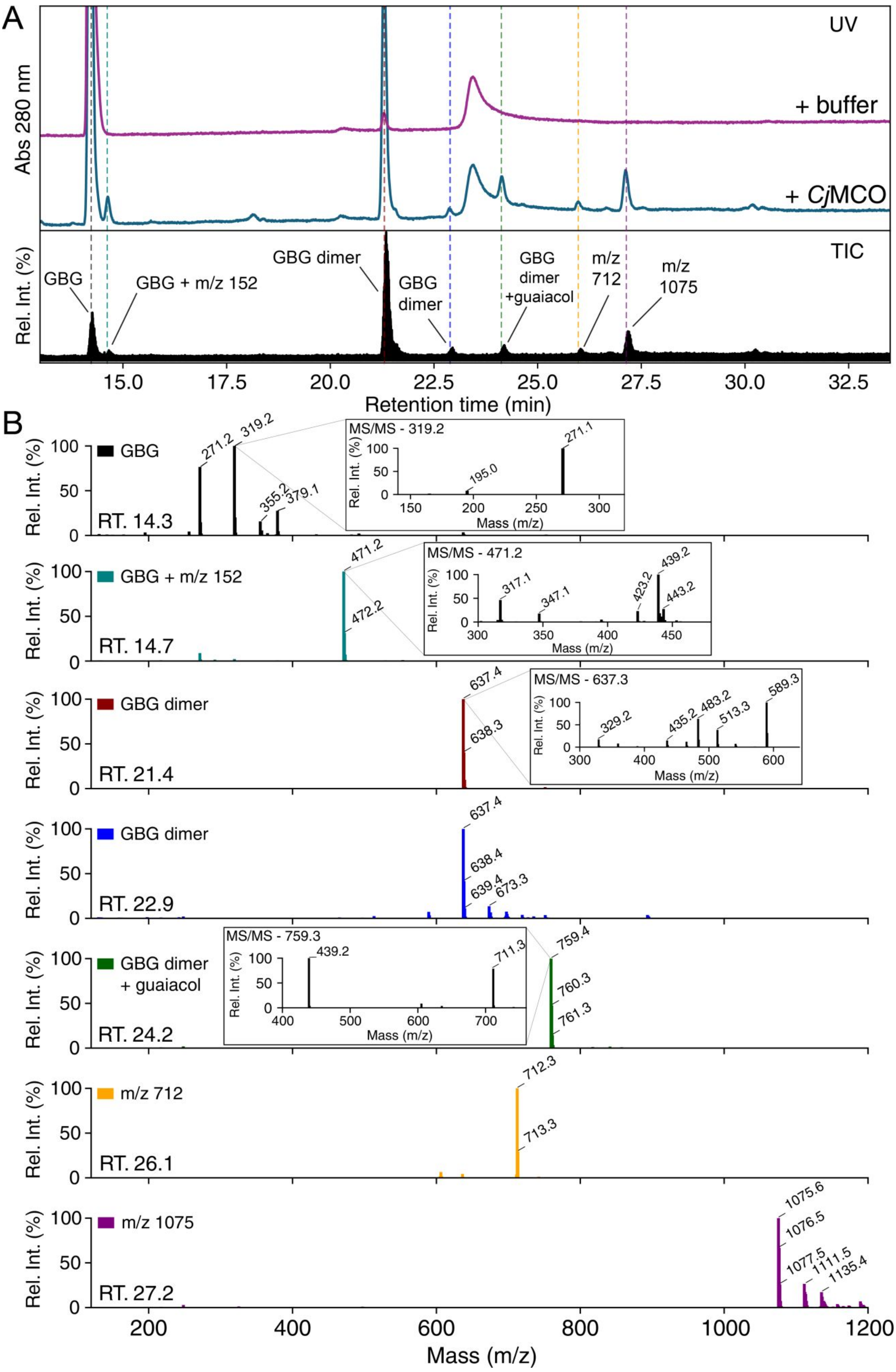

Figure S8. UHPLC-MS identification of products formed during oxidation of GBG by *Cj*MCO. **(A)** Overlay of UV chromatograms at 280 nm and total ion chromatogram (TIC) from UHPLC-MS

analysis in negative mode of GBG incubated with either buffer (purple trace) or Wt-*Cj*MCO enzyme (blue trace). The UV peak at RT 23 min is an injection-independent artifact also present in blank runs. **(B)** Negative mode full MS spectra of the seven peaks annotated in panel A, showing tentative product assignments: unreacted GBG (*m/z* 319.2, black), GBG + *m/z* 152 coupling product (teal), two chromatographically distinct GBG dimer isomers (red and blue), GBG dimer + guaiacol (green), and two unidentified higher-mass products at *m/z* 712 (yellow) and *m/z* 1075 (pink). Retention time (RT) where the MS spectrum was obtained is indicated bottom left in each spectrum. All ions are assumed to be $[M–H]^-$ species. Inserts show representative MS/MS fragmentation spectra for selected ions.

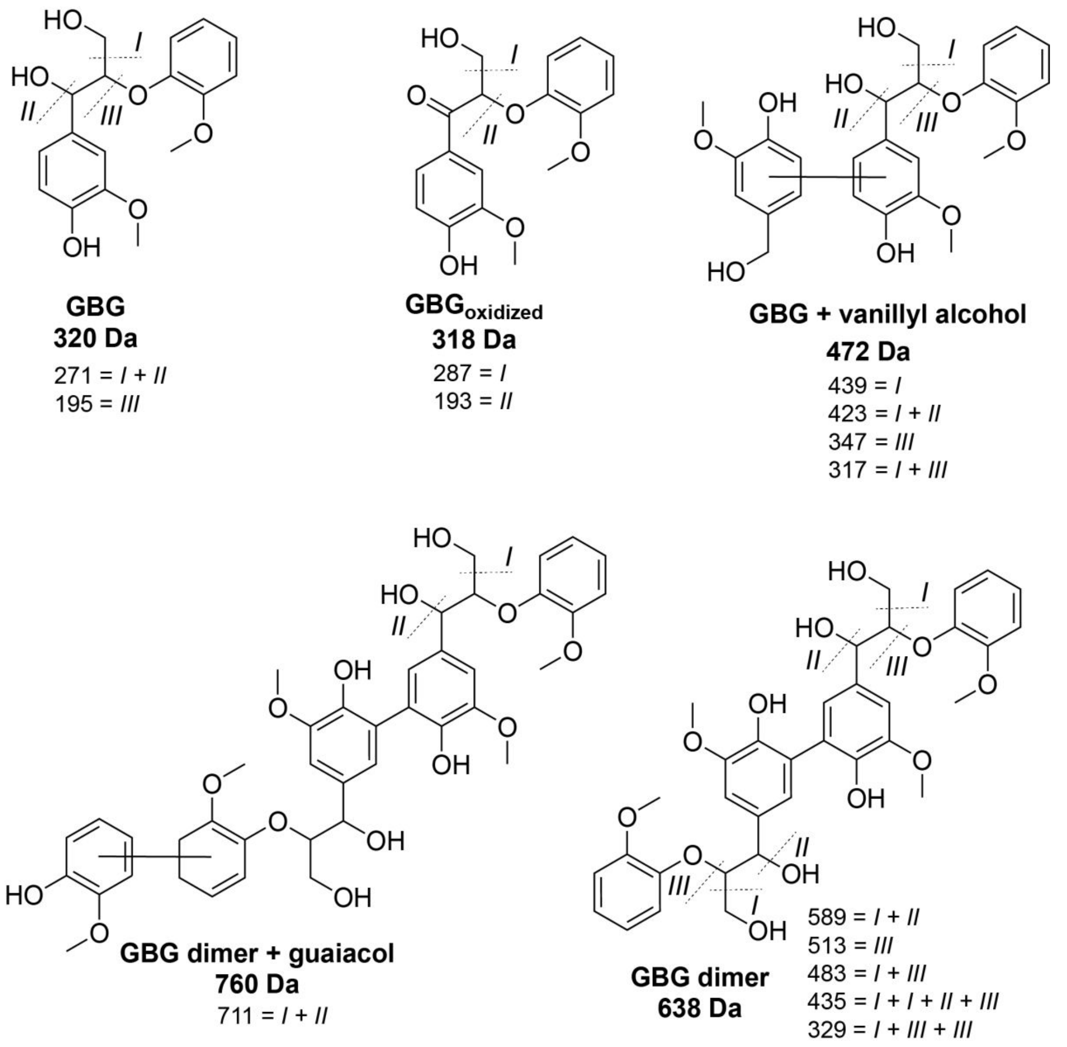

Figure S9. Tentative products resulting from oxidation of GBG by *Cj*MCO. Structures represent tentatively annotated products corresponding to masses observed in the UHPLC-MS mass spectra shown in **Figure S8** and the MALDI spectrum shown in **Figure 4** of the main manuscript. Dashed lines indicate proposed fragmentation sites based on the MS/MS spectra shown in **Figure S8**, and the adjacent Roman numerals correspond to the specific mass loss from the precursor ion *m/z*.

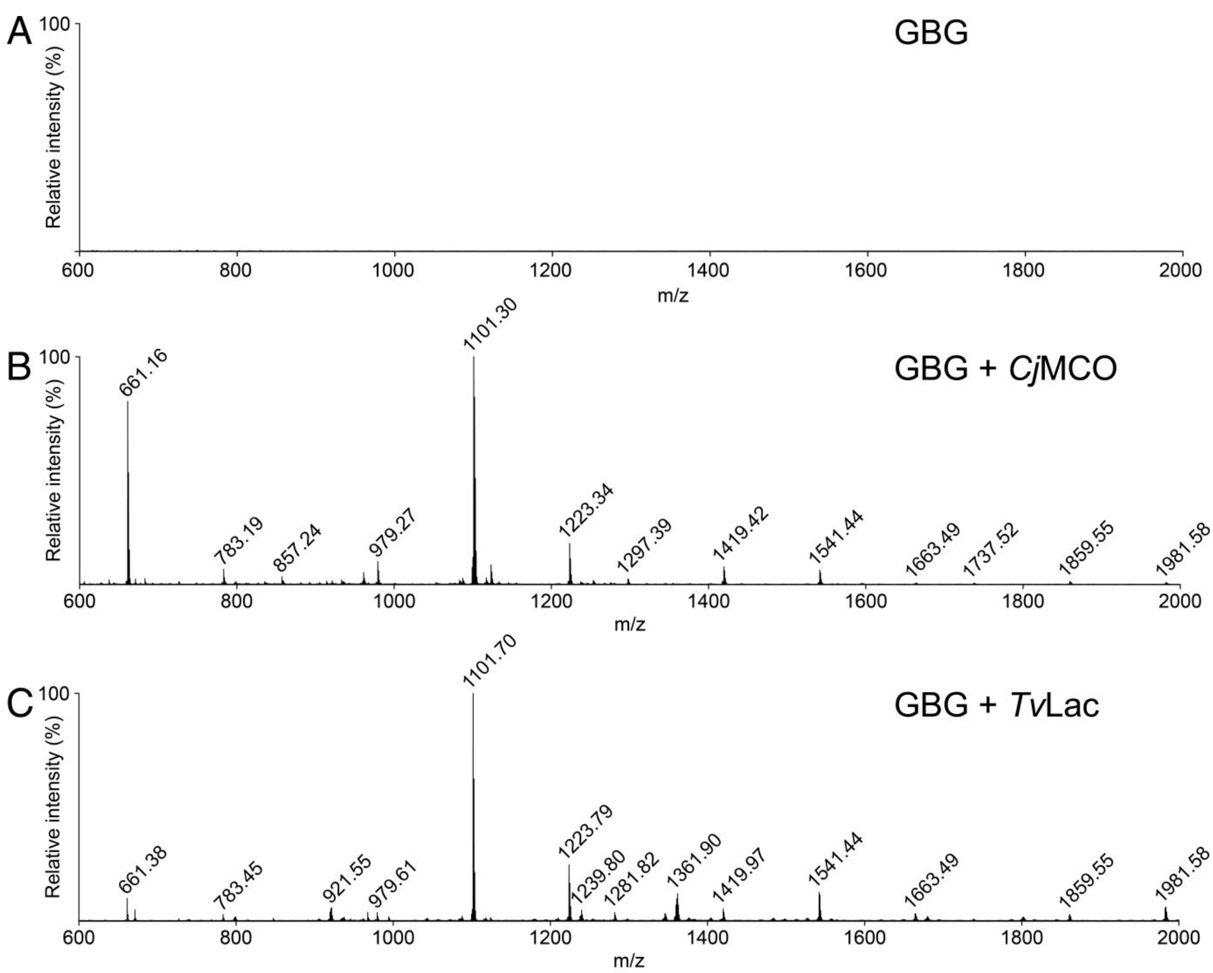

Figure S10. MALDI-ToF MS Spectra of GBG oxidized by *Cj*MCO and *Tv*Lac. The mass spectra reveal oligomeric products formed after incubating guaiacylglycerol-β-guaiacyl ether (GBG) with **(A)** no enzyme, **(B)** *Cj*MCO or **(C)** *Tv*Lac. Reactions were performed in 50 mM sodium phosphate buffer, pH 7.0, for 3 hours at 25 °C. See **Figure 4C** and **Table S3** for annotation.

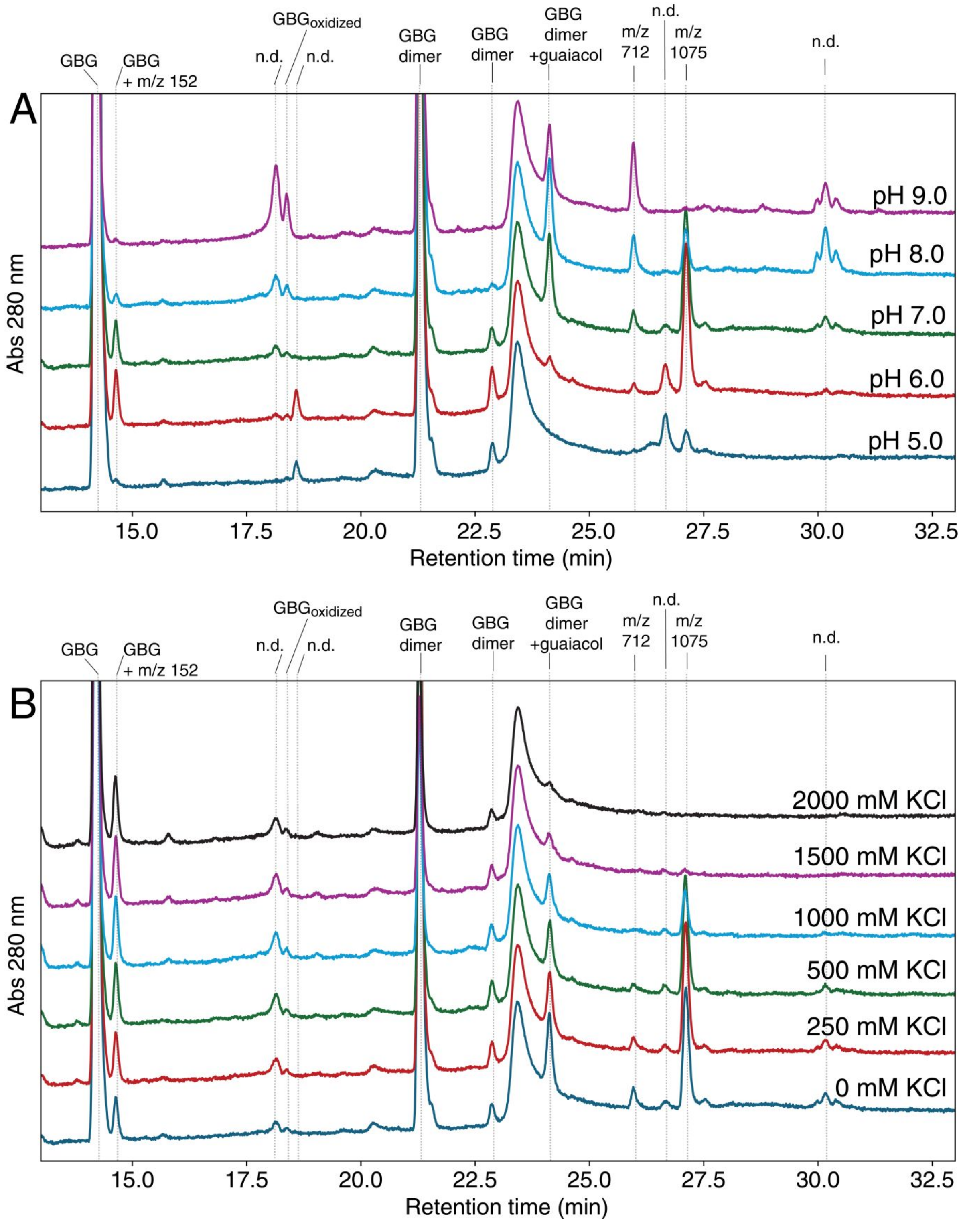

Figure S11. HPLC profiles showing product formation during oxidation of GBG by *Cj*MCO at varying pH and ionic strength. (A) Effect of pH on oxidation of GBG. The HPLC chromatograms show oxidation products after incubation of 200 µM GBG with 1 µM *Cj*MCO for 24 hours at 30°C. Reactions were performed in a combined buffer (20 mM sodium acetate, 20 mM sodium phosphate, and 20 mM Tris-HCl) adjusted to pH values ranging from 5.0 to 9.0. Peaks labeled "n.d." reflect products for which the identity was not determined. The peak at RT 23 min represents an injection-

independent artifact also observed in blank runs. **(B)** Effect of ionic strength on oxidation of GBG by *Cj*MCO at pH 7.0 (20 mM sodium acetate, 20 mM sodium phosphate, and 20 mM Tris-HCl as in panel A). The HPLC chromatograms show oxidation products after incubation of 200 μM GBG with 1 μM *Cj*MCO for 24 hours at 30°C. Reactions were performed with varying concentrations of KCl (0 to 2000 mM). The inset plot shows the conversion of GBG at varying KCl concentration. Annotation of peaks is the same as in panel A.

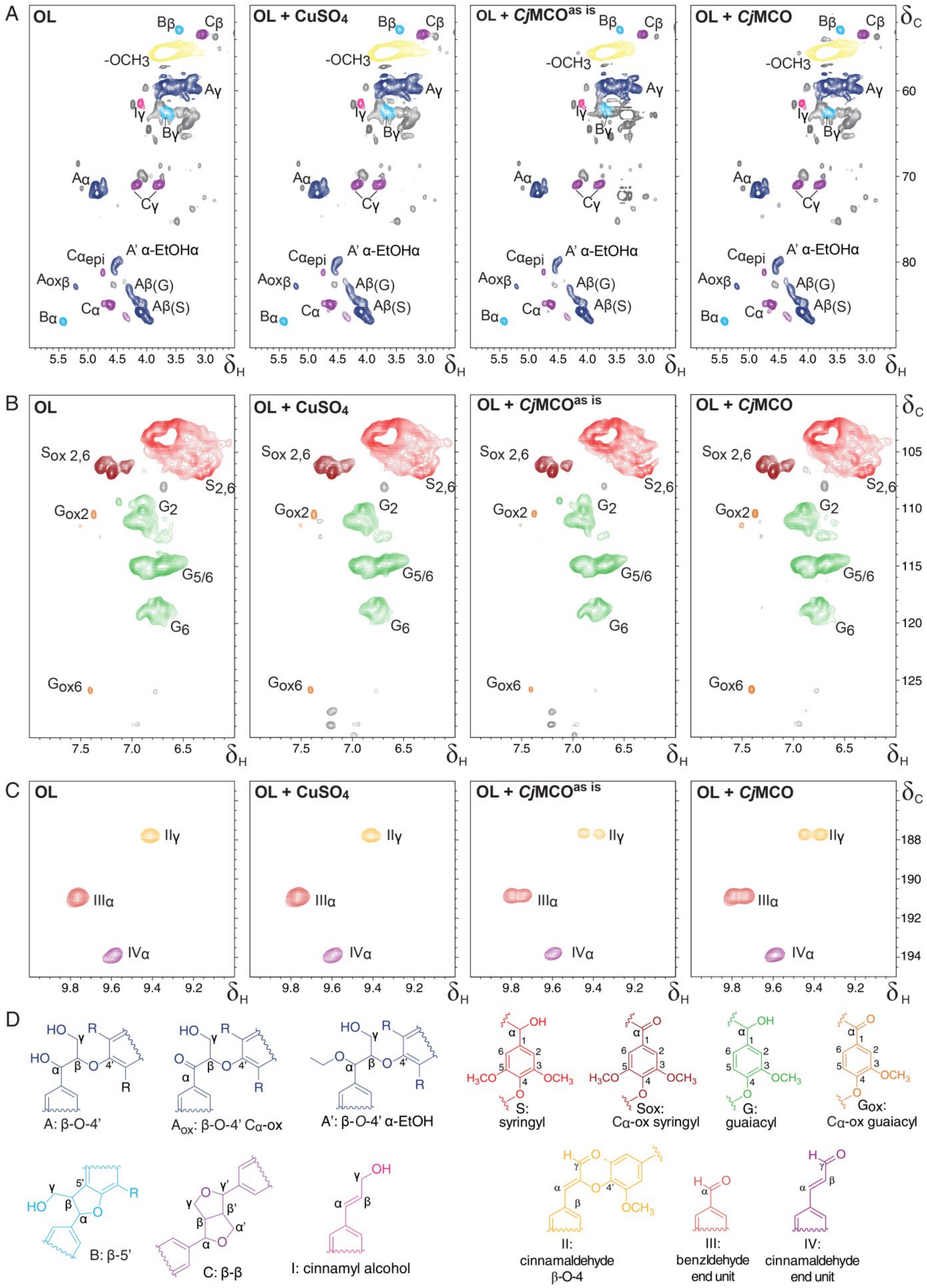

Figure S12. 2D-HSQC NMR spectra of organosolv lignin (OL). The spectra show organosolv lignin extracted from birch before incubation and after incubation with 30 µM $CuSO_4$, 5 µM *Cj*MCO$^{as\ is}$, or 5 µM *Cj*MCO. Reactions were carried out in 25% acetonitrile, 10 mM sodium phosphate buffer, pH 7.0,

and incubated at 150 rpm, and 25 °C. (**A)** Aliphatic, (**B)** aromatic, and (**C)** end-unit regions, are shown. The aliphatic and aromatic regions of spectra "OL" and "OL + CjMCO" are also shown in **Figure 6**. Annotations use subscripted numbers and Roman letters to specify the carbon atom from which the signal originates. **(D)** Annotated lignin substructures, with colors corresponding to signals in panels A, B, and C. "R" denotes either hydrogen or a methoxy group. Unassigned peaks are shown in gray.

Table S1. Copper content quantification of *Cj*MCO and *Cj*MCOΔ25–66 with and without copper reconstitution. The table shows the average number of copper atoms per protein molecule determined using the 2,2-biquinoline assay. *Cj*MCO and *Cj*MCOΔ25–66 without copper reconstitution is referred to "as is". Control analysis was performed with a 0.1 mM bovine serum albumin (BSA) solution supplemented with 0.4 mM $CuSO_4$ which is equivalent to four copper atoms per protein molecule. n.d. means not detected. Values are given as means ± standard deviations (n=3).

**Protein**	**Copper atoms per protomer**
*Cj*MCO	6.1 ± 0.1
*Cj*MCOΔ25–66	4.1 ± 0.2
*Cj*MCO^{asis}	n.d.
*Cj*MCOΔ25–66^{asis}	n.d.
0.1 mM BSA + 0.4 mM $CuSO_4$	3.92 ± 0.06

Table S2. Kinetic parameters for DMP oxidation by *Cj*MCO analyzed with different buffers, ionic strength and pH.

pH	Buffer	k_{cat} (s^{-1})	K_m (mM)	k_{cat}/K_m ($M^{-1}s^{-1}$)
4.0	50 mM sodium acetate	0.074 ± 0.004	2.4 ± 0.3	30 ± 4
4.5	50 mM sodium acetate	0.18 ± 0.02	2.1 ± 0.4	90 ± 20
5.0	50 mM sodium acetate	0.30 ± 0.02	2.9 ± 0.4	100 ± 20
5.5	50 mM sodium acetate	0.214 ± 0.006	2.4 ± 0.1	89 ± 6
5.5	50 mM MES	0.12 ± 0.02	6 ± 1	18 ± 5
6.0	50 mM MES	0.07 ± 0.01	3 ± 1	22 ± 8
6.5	50 mM MES	0.076 ± 0.008	1.9 ± 0.5	40 ± 10
6.5	50 mM MOPS	0.074 ± 0.004	3.7 ± 0.4	20 ± 2
7.0	50 mM MOPS	0.051 ± 0.007	1.5 ± 0.5	30 ± 10
7.5	50 mM MOPS	0.065 ± 0.007	1.8 ± 0.5	40 ± 10
7.5	50 mM TRIS	0.26 ± 0.02	1.9 ± 0.3	140 ± 20
8.0	50 mM TRIS	0.21 ± 0.01	1.4 ± 0.2	160 ± 20
8.5	50 mM TRIS	0.137 ± 0.007	1.1 ± 0.2	130 ± 20
9.0	50 mM TRIS	0.08 ± 0.01	0.7 ± 0.3	110 ± 40
5.0	50 mM Citrate	0.100 ± 0.008	2.5 ± 0.3	40 ± 6
5.0	50 mM Citrate + 100 mM KCl	0.032 ± 0.001	0.89 ± 0.09	35 ± 4
5.0	50 mM Citrate + 500 mM KCl	0.008 ± 0.000	0.750 ± 0.000	10.76 ± 0.00
5.0	500 mM Citrate	0.09 ± 0.01	3.4 ± 0.8	25 ± 7
6.0	50 mM Citrate	0.100 ± 0.008	3.3 ± 0.5	30 ± 5
6.0	50 mM Phosphate	0.165 ± 0.005	1.28 ± 0.09	130 ± 10
7.0	50 mM Phosphate	0.101 ± 0.001	0.91 ± 0.03	111 ± 4
7.0	50 mM Phosphate + 100 mM KCl	0.116 ± 0.002	0.66 ± 0.02	176 ± 6
7.0	50 mM Phosphate + 500 mM KCl	0.095 ± 0.001	0.29 ± 0.01	330 ± 10
7.0	500 mM Phosphate	0.143 ± 0.004	0.92 ± 0.04	155 ± 8
8.0	50 mM Phosphate	0.111 ± 0.002	0.57 ± 0.03	196 ± 9

Table S3. Compounds detected by UHPLC–MS after oxidation of GBG with *Cj*MCO. "+" indicates fragments covalently coupled to GBG.

Retention time (min)	Tentative annotation	Ionization	Observed/ exact mass	MS^2 fragments (relative intensity, %)
14.3	GBG	$[M-H]^-$	320.2/320.1	271 (100), 195 (8)
14.7	GBG + *net* vanillyl alcohol	$[M-H]^-$	472.2/472.2	439 (100), 443 (28), 440 (18), 423 (23), 347 (18), 317 (46)
18.3	$GBG_{oxidized}$	$[M-H]^-$	318.1/318.1	287 (100), 193 (60)
21.4, 22.9[a]	GBG dimer	$[M-H]^-$	638.4/638.2	589 (100), 513 (39), 483 (63), 465 (12), 435 (15), 329 (17)
24.2	GBG + *net* guaiacol	$[M-H]^-$	760.4/760.3	439 (100), 605 (9), 711 (79)

[a] Two chromatographically distinct isomers

Table S4. MALDI-TOF MS analysis of oligomeric compounds formed upon oxidation of guaiacylglycerol-beta-guaiacyl ether (GBG) by *Cj*MCO. The table presents the observed mass-to-charge ratio (*m/z*), the type of ion, and the tentative identity of selected detected compounds.

***m/z* observed**	**Ionization**	**Tentative annotation**
661.16	$[M+Na]^+$	GBG dimer
783.19	$[M+Na]^+$	GBG dimer + guaiacol
979.27	$[M+Na]^+$	GBG trimer
1101.30	$[M+Na]^+$	GBG trimer + guaiacol
1223.34	$[M+Na]^+$	GBG trimer + 2 guaiacol
1297.39	$[M+Na]^+$	GBG tetramer
1419.42	$[M+Na]^+$	GBG tetramer + guaiacol
1541.44	$[M+Na]^+$	GBG tetramer + 2 guaiacol

Table S5. Hydroxyl content (mmol/g) of birch organosolv lignin as determined by ^{31}P NMR. Aliphatic: Aliphatic OH groups, Benzoic acid: COOH groups in aromatic rings. S+G cond.: Condensed syringyl (S) + guaiacyl (G) units. G: Non-condensed guaiacyl units. Total phenolic: Sum of all phenolic hydroxyl groups (condensed + non-condensed)

	Aliphatic	Benzoic acid	S+G cond.	G	Total phenolic
**Birch OL**	1.6	0.18	0.85	0.32	1.17

Table S6. Semi-Quantitative HSQC NMR-derived parameters for organosolv lignin (OL). Reactions contained 10 mM sodium phosphate buffer, pH 7.0, 2.5 mg/mL OL and 5 µM *Cj*MCO/*Cj*MCO$^{as\ is}$ or 30 µM $CuSO_4$.

**Subunits (%)**	**OL**	**OL + *Cj*MCO**	**OL + CjMCO$^{as\ is}$**	**OL + $CuSO_4$**
G	20.9	18.3	19.5	19.1
G_{ox}	0.8	1.2	0.9	1.0
S	70.9	72.1	71.9	71.8
S_{ox}	7.4	9.5	8.6	9.2
S/G	3.61	4.13	3.89	3.99
**Interunit linkages (per 100 ar)**				
β-*O*-4 aryl ether	25.4	30.0	26.3	28.1
β-*O*-4 $C_{\alpha\text{-ox}}$	0.8	1.0	0.8	0.9
β-*O*-4 α-ethoxylated	5.4	5.9	5.0	5.5
β-5 phenylcoumaran	2.2	2.5	2.5	2.4
β-β resinol	4.2	4.7	4.4	4.5
β-β epiresinol	1.2	0.9	1.0	0.9
**End units (per 100 ar)**				
Cinnamyl alcohol	1.1	1.3	1.0	1.0
Cinnamaldehyde	1.0	1.2	1.0	1.0
Benzaldehyde	2.6	3.0	2.5	2.8
**Condensation (-)**				
G_2/Methoxyl	0.149	0.119	0.133	0.126

References

(1) Teufel, F.; Almagro Armenteros, J. J.; Johansen, A. R.; Gíslason, M. H.; Pihl, S. I.; Tsirigos, K. D.; Winther, O.; Brunak, S.; von Heijne, G.; Nielsen, H. Signalp 6.0 Predicts All Five Types of Signal Peptides Using Protein Language Models. *Nat. Biotechnol.* **2022**, *40* (7), 1023-1025.

(2) Bugg, T. D. The Chemical Logic of Enzymatic Lignin Degradation. *Chem. Commun.* **2024**, *60* (7), 804-814.

(3) Zhang, C.; Shen, X.; Jin, Y.; Cheng, J.; Cai, C.; Wang, F. Catalytic Strategies and Mechanism Analysis Orbiting the Center of Critical Intermediates in Lignin Depolymerization. *Chem. Rev.* **2023**, *123* (8), 4510-4601.
